# Dynamic Properties of the Alkaline Vesicle Population at Hippocampal Synapses

**DOI:** 10.1371/journal.pone.0102723

**Published:** 2014-07-31

**Authors:** Mareike Röther, Jan M. Brauner, Katrin Ebert, Oliver Welzel, Jasmin Jung, Anna Bauereiss, Johannes Kornhuber, Teja W. Groemer

**Affiliations:** Department of Psychiatry and Psychotherapy, University of Erlangen-Nuremberg, Erlangen, Germany; Centre national de la recherche scientifique, University of Bordeaux, France

## Abstract

In compensatory endocytosis, scission of vesicles from the plasma membrane to the cytoplasm is a prerequisite for intravesicular reacidification and accumulation of neurotransmitter molecules. Here, we provide time-resolved measurements of the dynamics of the alkaline vesicle population which appears upon endocytic retrieval. Using fast perfusion pH-cycling in live-cell microscopy, synapto-pHluorin expressing rat hippocampal neurons were electrically stimulated. We found that the relative size of the alkaline vesicle population depended significantly on the electrical stimulus size: With increasing number of action potentials the relative size of the alkaline vesicle population expanded. In contrast to that, increasing the stimulus frequency reduced the relative size of the population of alkaline vesicles. Measurement of the time constant for reacification and calculation of the time constant for endocytosis revealed that both time constants were variable with regard to the stimulus condition. Furthermore, we show that the dynamics of the alkaline vesicle population can be predicted by a simple mathematical model. In conclusion, here a novel methodical approach to analyze dynamic properties of alkaline vesicles is presented and validated as a convenient method for the detection of intracellular events. Using this method we show that the population of alkaline vesicles is highly dynamic and depends both on stimulus strength and frequency. Our results implicate that determination of the alkaline vesicle population size may provide new insights into the kinetics of endocytic retrieval.

## Introduction

Synaptic vesicle recycling is crucial for sustained neurotransmitter release [1]. Triggered by action potentials, synaptic vesicles fuse with the plasma membrane to release neurotransmitters into the synaptic cleft. This is followed by compensatory endocytic retrieval of membranes which is clathrin-mediated [2], [3].

Generally, the intravesicular pH is acidic in contrast to the extracellular fluid [4]. This proton gradient is maintained by the vesicular proton pump. For single synaptic vesicles, acidification time constants of 500 ms to 1 s have been reported [5]. Reacidification of synaptic vesicles is essential for the accumulation of neurotransmitters as transporters inside the synaptic vesicle membrane use the electrical and chemical gradient for intravesicular neurotransmitter concentration [3]. Consequently, upon compensatory endocytic retrieval, those vesicles that are internalized, but not yet reacidified, are captured in an alkaline state and form the “population of alkaline vesicles“ [6]. As reacidification occurs very fast after formation of a clathrin-coated vesicle [5]–[7], alkaline vesicles pinpoint synaptic vesicle scission which is an indispensable step upon endocytic retrieval [8].

Nevertheless, in contrast to exo- and endocytosis, the kinetics of the alkaline vesicle population is far less explored to date as intracellular events are difficult to visualize. Using repeated cycling of external pH, membrane scission has been shown at fibroblasts transfected with a pH-dependent fluorescent fusion protein containing transferrin receptor [8]. Another group used changes of external pH to monitor the kinetics of endocytosis while blocking reacidification with an inhibitor of the proton pump [2]. Additionally, in previous work, the kinetics of synaptic vesicle reacidification at hippocampal nerve terminals have been measured after short trains of stimulation that selectively mobilize the readily releasable pool, using pulse-pressure application of impermeant acid [6]. Furthermore, this method was utilized to analyze temperature dependence of the kinetics of synaptic vesicle reacidification [7]. However, using this „alkaline trapping“ method, it is only possible to estimate the dynamics of the population of alkaline vesicles, as fragmented data generated by repetitive stimulation were used. In addition, the dynamics of the alkaline vesicle population was selectively analyzed after short stimulation.

To date, real-time measurements of dynamic properties of alkaline vesicles at the neuronal synapse depending on stimulus strength and frequency are still lacking.

Therefore, in the present study we aimed to directly visualize intracellular events at the rat's hippocampal neuron and to provide time-resolved measurements of the dynamics of the alkaline vesicle population. Our data reveals that the alkaline vesicle population is not a static system, but changes its size in a highly dynamic manner during synaptic vesicle recycling, increasing and decreasing upon different stimulation paradigms. We found that the time constants for reacidification and endocytosis depend on the stimulation paradigms. Furthermore, we propose a mathematical model that takes into account the features of our measurement system (changing external pH values). We show that the experimentally determined effects are predicted by this model of decay of vesicles into and from the alkaline vesicle population if time constants measured here or found in other reports [2] are used.

## Materials and Methods

### Ethics statement

All animals were handled in accordance with good animal practice as defined by the guidelines of the State of Bavaria and all animal work was approved by the Kollegiales Leitungsgremium of the Franz-Penzoldt Zentrum, Erlangen (reference number TS-1/10).

### Cell culture and transfection

Hippocampal neuronal cultures were prepared from one to four days old Wistar rats (Charles River, USA) as described [9]. Briefly, newborn rats were sacrificed by decapitation in accordance with the guidelines of the State of Bavaria. Hippocampi were removed from the brain in ice cold Hank's salt solution, and the dentate gyrus was cut away. After digestion with trypsin (5 mg/ml) cells were mechanically triturated and plated in MEM medium, supplemented with 10% fetal calf serum and 2% B27 Supplement (all from Invitrogen, Taufkirchen, Germany). Neurons were transfected on DIV3 with synapto-pHluorin [4] under control of a synapsin promoter or with VGLUT1-pHluorin [10], [11] (kindly provided by Susan Voglmaier, Department of Psychiatry at University of California, San Francisco) under control of a beta-actin-promotor with CMV-enhancer, using a modified calcium phosphate method [12]. In brief, the culture medium was removed and replaced with Neurobasal A (Invitrogen, Taufkirchen, Germany). The calcium phosphate/DNA precipitate was allowed to form in BBS buffer (pH 7.05) for 30 minutes, then cells were transfected with the precipitate in Neurobasal A and incubated for 30 minutes prior to washing with HBSS (Invitrogen, Taufkirchen, Germany). Experiments were performed between 25 and 35 days *in vitro*.

### Live-cell fluorescence imaging

Experiments were conducted at room temperature on a Nikon TI-Eclipse inverted microscope equipped with a 60×, 1.2 NA water immersion objective and Perfect Focus System. Fluorescent dyes were excited by a Nikon Intensilight C-HGFI lamp (Neutral Density Filter 16) through excitation filters centred at 482 nm and 640 nm, using dichroic longpass mirrors (cut-off wavelength 500 nm and 650 nm). The emitted light passed emission band-pass filters ranging from 500 nm–550 nm and 660 nm–730 nm, respectively (Semrock, Rochester, NY) and was projected onto a cooled EM-CCD camera (iXonEM DU-885, Andor).

Cover slips were placed into a perfusion chamber (volume  = 500 µl) containing saline (144 mM NaCl, 2.5 mM KCl, 2.5 mM CaCl_2_, 2.5 mM MgCl_2_, 10 mM Glucose, 10 mM Hepes, pH 7.5). Synaptic boutons were stimulated by electric field stimulation (platinum electrodes, 10 mm spacing, 1 ms pulses of 50 mA and alternating polarity); 10 µM 6-cyano-7-nitroquinoxaline-2,3-dione (CNQX, Tocris Bioscience) and 50 µM DL-2-Amino-5-phosphonopentanoic acid (DL-AP5, Tocris Bioscience) were added to prevent recurrent activity.

### Experimental protocols

For colocalization experiments, images were acquired at 0.5 Hz frame rate with 500 ms exposure time. Neurons transfected with VGLUT1-pHluorin or synapto-pHluorin, respectively, were perfused with tyrode's solution of pH 7.5. At 30 seconds, neurons were stimulated with 200 action potentials at 10 Hz frame rate (200ap10Hz). At the end of the stimulus, perfusion was changed to tyrode's solution of pH 5.5.

For the anti-synaptotagmin-1-cypHer experiments, VGLUT1-pHluorin-transfected neurons were incubated for 30–60 minutes at 37°C with 0.6 µg of CypHer5E labeled anti-synaptotamin-1-antibody [13]–[15] (αSyt1-cypHer) (Synaptic Systems, Göttingen, Germany) in extracellular medium to allow intravesicular uptake of the antibody and thus staining of synaptic boutons. To identify active boutons, cells were stimulated with 200ap10Hz. Three subsequent images were acquired with 1000 ms exposure time.

For FM experiments, recycled synaptic vesicles were labeled with FM 1–43 [16] (Invitrogen, Taufkirchen, Germany). For staining, nerve terminals were loaded with 200ap20Hz using 2.5 µM FM 1–43. The dye was allowed to remain on the cells for 60 seconds after stimulation was finished to permit complete endocytosis, and was subsequently removed by a 7 minutes washout. The loaded boutons were then stimulated again with 200ap20Hz to evoke exocytosis. Images were recorded with 200 ms exposure time at 1 Hz frame rate.

For pH-cycling, an automated two-channel-perfusion system containing tyrode's solution of pH 7.5 and 5.5 was used. Images were acquired at 10 Hz frame rate with 100 ms exposure time. Neurons transfected with synapto-pHluorin or VGLUT1-pHluorin, respectively, were electrically stimulated according to the following protocol: For analysis of the influence of the number of action potentials at 20 Hz each with 200, 50, 100 and 400 pulses, and for analysis of the influence of the stimulus frequency with 200 pulses each at 20, 10, 40 and 80 Hz. The total protocol lasted 242 s with solution exchange every two seconds (Figure S1). At the beginning a short acid pulse was applied to test whether the perfusion system was in the right place. Afterwards, neurons were perfused for 26 seconds with tyrode's solution of pH 7.5 to have a stable baseline. 28 seconds after the beginning of the experiment pH-cycling was started. The stimulus trigger was set after 40 seconds.

For all experiments camera binning was 2×2 and resulting image stacks were converted into tagged image file format (TIFF).

### Image analysis

All image and data analysis was performed using custom-written routines in MATLAB (The MathWorks Inc., Natick). First, images were sorted into two stacks depending on the pH as described in the results. If the resulting stacks showed shifts, alignment with ImageJ was performed. Then, sets of images obtained upon the same pH phase were used to calculate an average image and the two mean fluorescence traces of pH 7.5 and 5.5 were built for each experiment. For further analysis, we measured fluorescence changes only in regions of interest (i.e. synaptic regions) of fixed dimensions. Thereby, average images of the sets of 3 images before and after stimulation were calculated, and a difference image of these average images was created. In this difference image, peak detection was performed, using a predefined threshold, a minimal and maximal limit for the size of the region and a laplacian shape. Afterwards, all mean fluorescence traces corresponding to the same stimulation paradigms were averaged to a common mean fluorescence trace and the baseline was subtracted.

### Statistical analysis

Statistical analysis was performed by MATLAB (The MathWorks Inc., Natick) using analysis of variance (Kruskal-Wallis-Test) if more than two groups were compared or unpaired t-test if two groups were compared. Error bars indicate SEM. In αSyt1-cypHer experiments, colocalization analysis was performed by Manders' colocalization [17]. For correlation analysis Pearson's coefficient was calculated.

### Simulation

The models we used for simulation are based on current literature [6], [7]. We modeled the exocytosis rate as constant during stimulation and directly proportional to the stimulus frequency so that cumulative exocytosis depended only on the number of action potentials [18]. Endocytosis and reacidification were assumed to be first-order processes so that their velocities were proportional to the surface pool or alkaline vesicle population, respectively.

We used two different models, an “ideal” model and a “realistic” model. The “ideal” model simply depicts the dynamics of the vesicle populations and was implemented as described above. The “realistic” model also reflects the fact that the processes during pH-cycling influence the vesicle population dynamics. For the “realistic” model, cycling between phases with perfusion of pH 7.5 and pH 5.5 was simulated. In the pH 7.5 phases, the vesicle populations behave as described above. In the pH 5.5 phases, endocytosis and reacidification are still processes with first order kinetics, but endocytosed vesicles are already acidic and do not contribute to the alkaline vesicle population.

Simulation was implemented in MATLAB (The MathWorks Inc., Natick). For details and equations please be referred to the supplementary methods.

### Deconvolution

**Figure 1:**
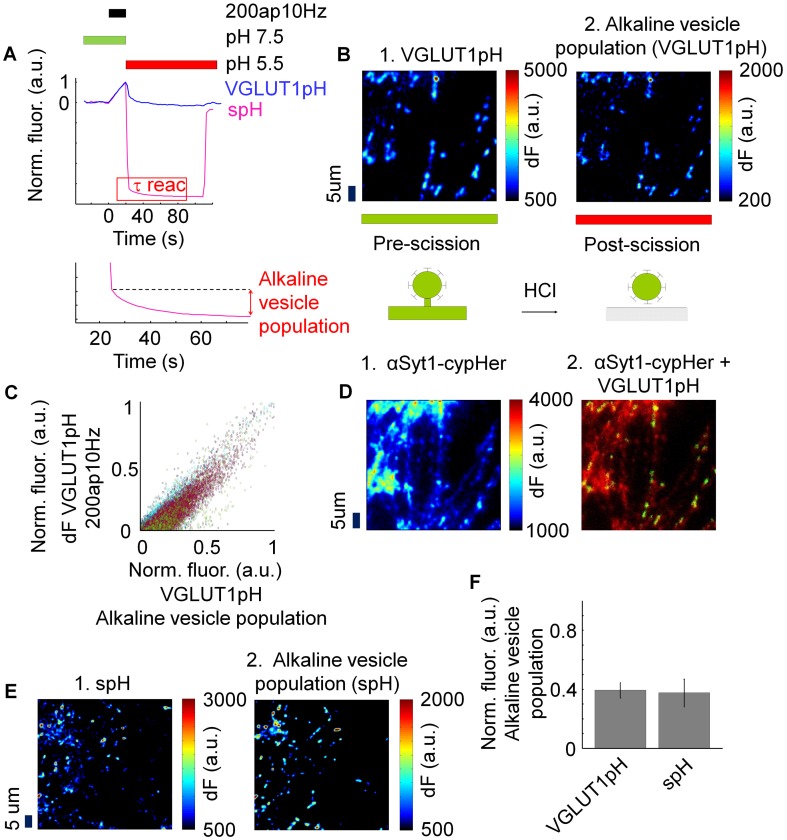
Determination of the alkaline vesicle population of synaptic vesicles with synapto-pHluorin and VGLUT1-pHluorin. (A) Mean time courses of normalized fluorescence of VGLUT1-pHluorin (VGLUT1pH) and synapto-pHluorin (spH) (N = 5). Bottom: Magnification of the red rectangle marked in (A). To determine the size of the alkaline vesicle population, difference of fluorescence was measured between end of surface fluorescence quenching and the end of reacidification when a stable baseline of fluorescence was reached. (B) 1. Difference image at pH 7.5 before and after stimulation of neurons transfected with VGLUT1-pHluorin. 2. Representative image of the alkaline vesicle population of neurons transfected with VGLUT1-pHluorin taken at pH 5.5 directly after stimulation. (C) Correlation between normalized fluorescence of the difference image before and after stimulation and normalized fluorescence of the alkaline vesicle population of neurons transfected with VGLUT1-pHluorin. (D) Representative images of neurons transfected with VGLUT1-pHluorin and incubated with αSyt1-cypHer. 1. One-color image (αSyt1-cypHer). 2. Dual-color-image (VGLUT1-pHluorin and αSyt1-cypHer) (Overlay). (E) 1. Difference image at pH 7.5 before and after stimulation of neurons transfected with synapto-pHluorin. 2. Representative image of alkaline vesicle population of neurons transfected with synapto-pHluorin taken at pH 5.5 directly after stimulation. (F) Fraction of the alkaline vesicle population depending on the fluorescent probe. There was no significant difference between the two fluorescent probes (VGLUT1-pHluorin: 39.38%, synapto-pHluorin: 37.69%, p>0.05, N = 5 each).

Deconvolution of the reacidification time course from the alkaline vesicle population dynamics and estimation of the endocytosis time course was performed as described earlier [6]. For this calculation it was assumed that endocytosis is the only input and reacidification the only output of the alkaline vesicle population. The reacidification kinetics was experimentally identified as a decaying exponential function with the time constant for reacidification (τ reac) (Figure 1 (A), Tables 1 and 2). This allowed for the calculation of the time course of endocytosis for various stimuli, assuming reacidification to be variable with regard to the stimulus condition. The endocytosis kinetics was calculated by deconvolution of the reacidification kinetics from the alkaline vesicle population time course as follows:

**Table 1:** The time constant for reacidification decreases with rising action potential number.

Action potential number	τ reac (s)
50ap20Hz	23.32±2.56 SEM (n = 6)
100ap20Hz	13.19±1.84 SEM (n = 6)
200ap20Hz	10.81±1.12 SEM (n = 11)
400ap20hz	9.31±0.51 SEM (n = 5)

**Table 2:** The time constant for reacidification increases with rising action potential frequency.

Action potential frequency	τ reac (s)
200ap10Hz	8.98±2.03 SEM (n = 5)
(200ap20Hz	10.81±1.12 SEM (n = 11))
200ap40Hz	12.28±1.83 SEM (n = 5)
200ap80hz	17.91±3.04 SEM (n = 5)

The mean alkaline vesicle population time course for every stimulus condition was fitted with an arbitrary alpha-function:
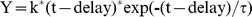
where *k* is a scaling factor, t is the time of the start after stimulus and delay is a time offset term.

Deconvolution was performed on the resulting smoothed function instead of the raw data, in order to minimize noise. The deconvolution was calculated by dividing the fast Fourier transform of the alkaline vesicle population kinetics by the fast Fourier transform of the reacidification kinetics and applying the reverse fast Fourier transform on the result. The resulting time course was fitted with an exponential decay function to obtain the time constant for endocytosis (τ endo). The algorithm was implemented in MATLAB (The MathWorks Inc., Natick).

## Results

To analyze the dynamics of the alkaline vesicle population we decided to use the fluorescent probe synapto-pHluorin which is a well-established vesicular marker and often used for the measurement of exo- and endocytosis [4], [19], [20]. Synapto-pHluorin is a pH-sensitive mutant of green fluorescent protein tagged to the luminal site of the synaptic vesicle transmembrane protein synaptobrevin-2 [4], [21]. In previous work it was found that synapto-pHluorin has a surface pool of 10–15% [4] or up to 24% [2]. As overexpression of pHluorin-tagged synaptic vesicular proteins might cause an artificial surface pool, the use of synapto-pHluorin as an optical probe has often been questioned [22], [23]. However, recent studies have shown that synapto-pHluorin participates in synaptic vesicle recycling [19], [24]. In addition, lateral movement of synapto-pHluorin was observed in previous studies [2]. Nevertheless, performing pH-cycling, fluorescence decay caused by lateral diffusion would be prevented by surface quenching [6]. In our laboratory synapto-pHluorin has been used in many studies and turned out to be a reliable marker for the measurement of exo- and endocytosis.

### Determination of the alkaline vesicle population of synaptic vesicles with synapto-pHluorin and VGLUT1-pHluorin

Nevertheless, as a first step we sought to test whether synapto-pHluorin is also a suitable fluorescent probe for the detection of the alkaline vesicle population.

Generally, if a fluorescent probe was a convenient marker for the detection of the alkaline vesicle population, the amount of vesicles detected in the alkaline state should be the same when measured with another fluorescent probe. With this aim, experiments with two different overexpression markers, synapto-pHluorin and VGLUT1-pHluorin, were performed. VGLUT1-pHluorin is a pH-dependent GFP-variant tagged to the vesicular glutamate transporter VGLUT1 [11]. In contrast to synapto-pHluorin, VGLUT1-pHluorin has a very small surface pool measuring 2–3% of the total marker population [10], which makes it unsuitable for pH-cycling experiments as pH phases can hardly be determined automatically from the time course itself (Figure S2, Text S1). However, static measurements are not hindered by the small surface pool of VGLUT1. We thus chose to test whether the alkaline population of synaptic vesicles was depicted similarly by VGLUT1-pHluorin and synapto-pHluorin even if recent data already indicated that both fluorescent probes do not perturb presynaptic function [10], [11], [25] and that pHluorins reliably record all fusion events [2].

First, it was interesting to compare the characteristics of both overexpression markers under electrical stimulation. Consequently, live-cell imaging was performed and neurons were electrically stimulated with a moderate stimulus of 200 action potentials at 10 Hz (200ap10Hz) according to the protocol shown in Figure 1 (A). As both pHluorins recycle within the synaptic vesicle membrane, their time courses of fluorescence indicate exocytosis and compensatory endocytosis [4]. For analysis, five independent experiments with each fluorescent probe were performed and all utilized.

At the beginning, neurons were constantly perfused with tyrode's solution of pH 7.5 (Figure 1 (A)). Under these conditions, only pHluorin-molecules resting at the membrane surface are fluorescent. At 30 seconds, neurons were stimulated with 200ap10Hz. Due to stimulation-induced exocytosis, intravesicular pHluorin-molecules come into contact with the extracellular fluid, and fluorescence rises [4]. At the end of the stimulation, external pH was changed to 5.5. Fluorescence then decreased due to the following reasons: First, surface fluorescence is quenched [4], [6], [8]. Notably, surface fluorescence of synapto-pHluorin turned out to be much greater than of VGLUT1-pHluorin [10]. Second, compensatory endocytosis with subsequent reacidification of recently endocytosed synaptic vesicles takes place [4], [6]. Those vesicles that are endocytosed, but not yet reacidified. are alkaline vesicles and belong to the alkaline vesicle population [6]. In these experiments, alkaline spots were visible as fluorescent spots that resisted the acidic extracellular pH.

If the fluorescent, acid-resistant spots that were detected by both fluorescent probes were indeed alkaline vesicles which are supposed to occur upon compensatory endocytosis, they should appear at regions where exocytosis takes place. Consequently, as a next step, colocalization between VGLUT1-pHluorin positive spots responding to the stimulus and alkaline spots was investigated. At extracellular pH 7.5, VGLUT1-pHluorin molecules that came in contact with the alkaline extracelluar fluid upon exocytosis are fluorescent (Figure 1 (B 1)). Due to changing extracellular pH to 5.5, surface fluorescence is quenched, whereas VGLUT1-pHluorin molecules in alkaline synaptic vesicles are still fluorescent and can be selectively measured (Figure 1 (B 2)).

To determine the extent of colocalization of VGLUT1-pHluorin positive spots responding to the stimulus and alkaline spots, Manders' coefficients were calculated [17]. About 78.9%±1.99% S.E.M. of VGLUT1-pHluorin positive spots responding to the stimulus colocalized with alkaline spots (M1 = 0.79), whereas only 65%±15.37% S.E.M. of alkaline spots colocalized with VGLUT1-pHluorin positive spots responding to the stimulus (M2 = 0.65). Correlation analysis was performed with Pearson's coefficient (Mean Pearson's coefficient: 0.84±0.02 SEM (coincidence: 7,05e -10)) (Figure 1 (C)).

In conclusion, the majority of alkaline spots seem to appear at VGLUT1-pHluorin positive regions where exocytosis takes place. Therefore, we conclude that these “alkaline spots” represent indeed alkaline synaptic vesicles. Even though, there were alkaline spots that did not colocalize.

If alkaline vesicles colocalized with VGLUT1-pHluorin positive regions where exocytosis takes place, they must appear at active synapses. To analyze this issue, VGLUT1-pHluorin-transfected cells were incubated for one hour with αSyt1-cypHer, an anti-Synaptotagmin-1 antibody labeled with the pH-dependent cyanine dye variant CypHer 5 [13]–[15], before execution of the experiments. αSyt1-cypHer has a pH-dependency which is inverse to that of VGLUT1-pHluorin so that it becomes fluorescent in an acidic environment [14]. Additionally, these fluorescent probes have different wavelengths, which enables for dual-color-microscopy. After electrical stimulation to provoke exocytosis and compensatory endocytosis, images of αSyt1-cypHer positive spots in the same region that was used for the experiment were acquired (Figure 1 (D)). Manders' colocalization revealed that 93.51% of the VGLUT1-pHluorin positive spots responding to the stimulus at pH 7.5 colocalized with αSyt1-cypHer positive spots (M2 = 0.92). Due to the high colocalization of VGLUT1-pHluorin positive spots responding to the stimulus with αSyt1-cypHer positive spots, these data indicate that alkaline vesicles appear with high probability at functional presynaptic boutons. In contrast to that, only 17.61% of αSyt1-cypHer positive spots colocalized with VGLUT1-pHluorin positive spots (M2 = 0.18). This is readily explained by the transfection rate of pHluorins, which is supposed to be 2–20 transfected neurons per cover slip [20].

As a next step, colocalization coefficients of synapses defined with synapto-pHluorin and alkaline spots were analyzed (Figure 1 (E)). Manders' colocalization revealed that about 81%±10.78% S.E.M. of the synapto-pHluorin positive regions responding to the stimulus colocalized with alkaline spots (M1 = 0.81) whereas only 49%±9.70% S.E.M. of alkaline spots colocalized with synapto-pHluorin positive regions responding to the stimulus (M2 = 0.49).

In conclusion, there was no significant difference in colocalization of alkaline vesicles measured with VGLUT1-pHluorin and synapto-pHluorin (M1: p = 0.86, M2: p = 0.40).

In summary, our data indicate that alkaline vesicles preferentially appear at synaptic regions. Nevertheless, there were alkaline vesicles that did not colocalize. This may be due to the fact that some synapses were not detected during analysis because of low response to stimulation. On the other hand, it was shown in earlier publications that endocytosis is associated with the movement of clathrin out of the synapse [2]. Therefore, it could thus be possible that alkaline vesicles do not exclusively appear at functional presynaptic boutons.

Finally, the size of the alkaline vesicle population was measured at neurons transfected with synapto-pHluorin in comparison to neurons transfected with VGLUT1-pHluorin. We supposed that both fluorescent probes should label the same amount of the alkaline vesicle population if alkaline vesicles were selectively visualized. To obtain a measure for the size of the alkaline vesicle population, difference of fluorescence was measured between the end of surface fluorescence quenching and the end of reacidification when a stable baseline of fluorescence was reached. (Figure 1 (A), bottom). Using this method, the size of the population of alkaline (immediately after stimulation with 200ap10Hz) turned out to be 39.38%±0.05% S.E.M. at neurons transfected with VGLUT1-pHluorin and 37.69%±0.09% S.E.M. at neurons transfected with synapto-pHluorin (Figure 1 (F)). There was no significant difference in the size of the alkaline vesicle population (p>0.05).

Furthermore, when analyzing synaptic vesicle recycling with the use of pHluorins upon epifluorescence microscopy, the effect of photobleaching should be considered.

In general, the fluorescence signal that can be measured when performing epifluorescence microscopy with pHluorins consists of the fluorescence emitted by the pHluorin (depending on the pH value) as well as of background fluorescence unrelated to the pHluorin (autofluorescence). The fluorescence emitted by the pHluorin thereby depends on the pH value: As shown in previous work [4], the fraction of ecliptic pHluorin molecules in the deprotonated state determines the fluorescence signal amplitude. Thereby, the increase in fluorescence for each vesicle fusing with the plasma membrane will be 20-fold upon external pH change from 5.5 to 7.5 [4]. Nevertheless, upon pH 5.5, there is still some fluorescence measurable.

Apart from that, the fluorescence measured at a certain point of time depends on the inside/outside ratio of the pHluorin. As up to 24% of the synapto-pHluorin molecules are resting at the surface membrane [2], the measured fluorescence consists of 24%×1 (surface pool)+76%×1/20 (quenched fraction). Therefore, about 86% of the measured fluorescence consists of fluorescence related to the surface pool. On the other hand, as VGLUT1-pHluorin has only a small surface pool of 2–3% [10], the measured fluorescence consists of 3%×1 (surface pool) +97%×1/20 (quenched fraction). Therefore, only about 38% of the measured fluorescence consists of fluorescence related to the surface pool. Consequently, the amount of pHluorin-background fluorescence, which is determined by the quenched fraction, is much higher for VGLUT1-pHluorin at synapses than for synapto-pHluorin. Photobleaching always affects the same part of the surface pool as well as of the quenched fraction, but is related to the amount of pHluorin molecules. Thus, in absolute terms, from the surface pool more synapto-pHluorin molecules than VGLUT1-pHluorin molecules will be affected by photobleaching, but background-bleaching or baseline-bleaching will be more prominent in the case of VGLUT1-pHluorin.

In summary, the data presented here shows that the alkaline vesicle population can be selectively visualized using both fluorescent markers. Alkaline vesicles colocalize to a high degree with regions where exocytosis takes place, and the majority of the alkaline vesicle population appears at synapses. In conclusion, VGLUT1-pHluorin as well as synapto-pHluorin are convenient fluorescent probes for the detection of the alkaline vesicle population at the neuronal synapse. Nevertheless, due to the small surface fraction of VGLUT1-pHluorin [10] which makes it unsuitable for automatic analysis (Figure S2, Text S1), we decided to rely on synapto-pHluorin for the following experiments.

### pH-cycling: Continuous monitoring of the alkaline vesicle fraction by alternated superfusion with different pH solutions

With the previous data, it was possible to visualize the alkaline vesicle population with a fluorescent probe at a particular point of time. However, to be able to analyze the dynamics of the alkaline vesicle population, another approach had to be established: Performing fast perfusion pH-cycling with solution exchange from pH 7.5 to pH 5.5 every two seconds, neurons were electrically stimulated and intracellular events were selectively visualized. The images captured at the two pH values were grouped into separate movies which were analyzed using custom-written program routines in MATLAB (The MathWorks Inc., Natick). After sorting the images according to different pH values, the alkaline vesicle population was selectively measured at images taken at pH 5.5. In contrast to previous work, where fragmented data generated by repetitive stimulation under pulse-pressure application of impermeant acid were used [6], pH-cycling enabled us to provide time-resolved measurements of the dynamics of the alkaline vesicle population at the neuronal synapse during a single stimulation.

**Figure 2:**
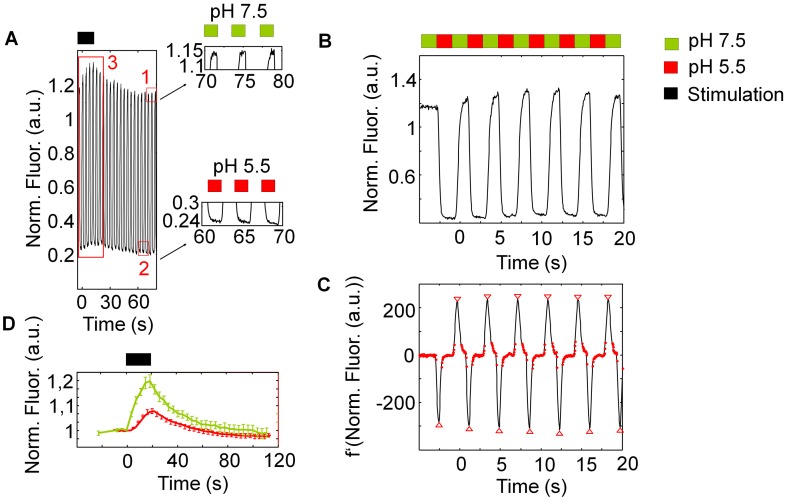
Experimental protocol and analysis procedure. (A) During rapid pH-cycling from pH 7.5 to 5.5 extracellular the fluorescence increases or decreases due to extracellular pH-change. These fluorescence-changes are superimposed by the exocytosis and endocytosis signal. (B) Magnification of the fluorescence trace in (A). For further analysis, images were divided into two series depending on the pH value. (C) Difference function (f′) of the respective fluorescence trace, where the minima and maxima (red triangles) serve as detection-markers for switching between the two different pH-phases. Values below a predefined threshold (red points) are analyzed as phase with pH 7.5 and as phase with pH 5.5, respectively. (D) Corresponding fluorescence traces of the signal at pH 7.5, which represents the kinetics of exocytosis and endocytosis, and at pH 5.5, which represents the kinetics of the alkaline vesicle population.

In Figure 2, the experimental protocol and analysis procedure are shown in detail. Neurons were electrically stimulated while cycling the extracellular pH. During the rapid change of extracellular pH from 7.5 to 5.5 and vice versa the fluorescence increased or decreased (Figure 2 (A)). Nevertheless, these fluorescence-changes were superimposed by the exocytosis and endocytosis signal. Due to stimulation, an increase of fluorescence could be observed at the images taken at pH 7.5 as well as pH 5.5 [4], [25]. The fluorescence trace obtained at the images taken at pH 7.5 corresponds to the time course of vesicles undergoing exo- and endocytosis whereas the fluorescence trace obtained at the images taken at pH 5.5 corresponds to the time course of the alkaline vesicle population. To be able to analyze the fluorescence traces separately, images had to be divided into two stacks depending on the pH. As it was not possible to define threshold values at the original fluorescence trace, separation had to be accomplished in an alternative way: The original fluorescence trace was characterized by the recurrent point of inflection at fluorescence increase from pH 5.5 to 7.5 and fluorescence decrease vice versa (Figure 2 (B), magnification of the big red rectangle marked in Figure 2 (A)). These points of inflection could be detected as minima and maxima in the first derivative of the original fluorescence trace (Figure 2 (C)). Using an automated detection program the point of time of the minima and maxima could be measured. Therefore, in our study, separation of the images was accomplished by definition of the point of time of the minima and maxima in the first derivative. As a result, we obtained two separate fluorescence traces which corresponded to the two different pH values (Figure 2 (D)). The processes that underlie both fluorescence traces can only be seen as a net change of fluorescence due to pH changes. Therefore, the fluorescence trace obtained upon pH 7.5 reflects (alkalisation upon) exocytosis and (reacidification following) endocytosis whereas the fluorescence trace obtained upon pH 5.5 mirrors endocytosis as well as the decay of the alkaline vesicle population into acidic vesicles.

Thereby, the initial fluorescence value at pH 5.5 is related to 1/20 fluorescence emitted by the pHluorin plus background fluorescence unrelated to the pHluorin. However, as the fluorescence time course been measured was sensible to photobleaching (Figure 2 (A+D)), we assume that the initial fluorescence value at pH 5.5 mainly consists of fluorescence emitted by the quenched pHluorin-population rather than of autofluorescent background.

In conclusion, here we present a novel methodical approach which takes into account that the intravesicular marker will stay fluorescent after application of impermeant acid. This method enables to precisely determine the dynamics of the alkaline vesicle population size and to analyze this indispensable step upon clathrin-mediated endocytosis.

### Influence of low extracellular pH on synaptic vesicle recycling

For the interpretation of measurements of synaptic vesicle recycling at varying external pH values, the influence of the external pH itself on synaptic vesicle turn-over must be known. A suppressive, but reversible effect on endocytosis in lizard neuromuscular junctions was reported [26]. To determine the influence of low external pH on synaptic vesicle recycling, we performed experiments using the established styryl dye FM 1–43 [16]. As depicted in Figure S3 (A), untransfected neurons were stimulated with 200ap20Hz, an intermediate, physiological stimulus, to permit loading of the styryl dye at pH 5.5 or at pH 7.5, respectively. At 30 seconds, after wash-out, unloading was performed using the same stimulus. Indeed, loading of FM 1–43 was reduced upon external pH 5.5 (Figure S3 (B+C)); however, this effect turned out to be insignificant (pH 7.5 (N = 6), pH 5.5 (N = 4), p = 0.092). When releasing FM-stained vesicles, we found that released vesicles previously loaded at pH 5.5 were significantly fewer than if they had been loaded at pH 7.5 (pH 7.5 (N = 6), pH 5.5 (N = 3), p = 0.013 (Figure S3 (D+E)). In summary, in line with previous work, our data show that long application of pH 5.5 reduces endo- and exocytosis at the hippocampal synapse. Nevertheless, we prove that this suppression is reversible upon pH 7.5. Therefore, we would suppose that upon pH-cycling, synaptic vesicle recycling may be slightly influenced by the short pH 5.5 phases, but as this is the only method to analyze the alkaline vesicle population to date, we argue that this effect has to be considered, but must be tolerated.

### Influence of acid and alkaline phase length on the determined amount of alkaline vesicles

Upon pH-cycling, only those synaptic vesicles that have been endocytosed upon external pH 7.5 will be detected during the acidic period due to their alkaline state. Therefore, it is likely that the amount of alkaline vesicles is underestimated if acid pulses are applied. If this is the case, the amount of alkaline vesicles must be sensitive to shifts in the ratio between acid and alkaline perfusion interval lengths. However, as we were not satisfied with a mere argumentation, we probed the influence of the variation of cycling periods on the relative amounts of detected alkaline vesicles (Figure S4). We found that when cycling with 2 seconds acid solution and 4 seconds alkaline solution, the population of alkaline vesicles was by a factor of 3 larger than if the relation was the opposite (N = 6, n = 274) (Figure S4 (E)).

In conclusion, these data indicate that indeed the relative size of the alkaline vesicle population depends on the perfusion interval length, leading to an underestimation of the number of detected alkaline vesicles. Consequently, concerning quantitative analysis of the alkaline vesicle population using pH-cycling, the following limitations have to be done: First, quantitative analysis has to be based on the relative size of the alkaline vesicle population, depending on the number of vesicles being exocytosed. Second, for comparative analysis only neurons from the same charge should be used, and experiments should be performed consecutively at the same region to have the same synapses. Third, comparative analysis should only be performed if the same pH phase lengths are used. We are aware that these limitations are extensive, but again we are of the opinion that as long as no better instrumental approach exists to monitor alkaline vesicles, these limitations have to be tolerated.

### The relative size of the alkaline vesicle population depends on the number of action potentials

Based on the data presented in Figure 2, we were now able to perform pH-cycling based simultaneous measurements of the alkaline vesicle fraction and the surface fraction. This enabled us to analyze the dynamics of the size of the alkaline vesicle population under different stimulation paradigms. In the following, we define as “relative size of the alkaline vesicle population” the ratio of the alkaline vesicle population and the amount of exocytosed vesicles.

First, we set out to analyze the influence of the number of action potentials on the size of the alkaline vesicle population as different stimulation strength and frequency are supposed to release vesicle pools with different release probabilities [27], [28]. While performing pH-cycling, neurons were electrically stimulated with 50ap20Hz, 100ap20Hz, 200ap20Hz and 400ap20Hz (N = 7, n = 374). As it has been shown that synapses at hippocampal neurons are very heterogeneous and that smaller and larger synapses seem to have differing release probabilities [29], [30], all experiments were consecutively performed at the same region to have the same synapses for quantitative analysis. Even though, recent data show that the speed of endocytosis does not differ between synapses with lower and higher release probability [2]. Between two subsequent experiments it was waited for 3 minutes to allow neurons to recover. All experiments were performed according to the same protocol which is depicted in Figure S1.

**Figure 3:**
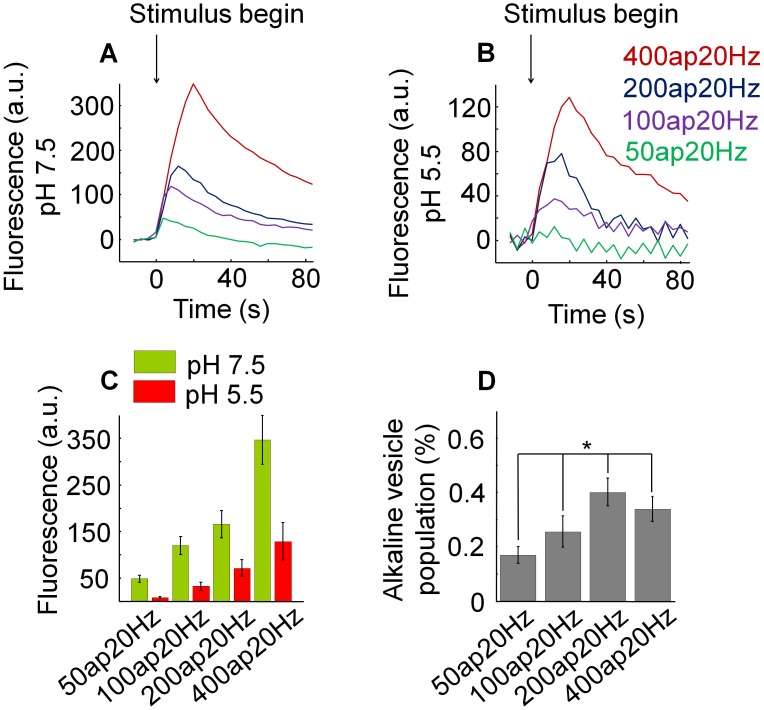
The relative size of the alkaline vesicle population depends on the number of action potentials. (A) Mean fluorescence profiles at pH 7.5 for different number of action potentials at 20 Hz each: 50, 100, 200 and 400 (N = 7, n = 374). Increasing the action potential number caused a fluorescence increase (p<0.001). (B) Mean fluorescence profiles at pH 5.5 for different number of action potentials at 20 Hz each: 50, 100, 200 and 400 (N = 7, n = 374). Increasing the number of action potentials caused a fluorescence increase (p<0.005). (C) Mean fluorescence changes at pH 5.5 and pH 7.5 induced by different numbers of action potentials at 20 Hz each: 50, 100, 200, 400 (N = 7, n = 374). To estimate the maximal alkaline vesicle population size with respect to the recycling pool, corresponding amplitudes of fluorescence measured at maximum under pH 5.5 and pH 7.5 were divided by each other. (D) Relative size of the alkaline vesicle population depending on the action potential number (N = 7, n = 374). The relative size of the alkaline vesicle population significantly depends on the number of action potentials (p<0.005).

As shown in Figure 3 (A), at the images taken at pH 7.5 increasing the action potential number caused a significant increase of the amplitude of the fluorescence trace at maximum due to the rise of the number of exocytosed vesicles (p<0.001). At the images taken at pH 5.5 the amplitude of the fluorescence trace at maximum also significantly increased with higher number of action potentials (p<0.005) (Figure 3 (B)). The pH 5.5 fluorescence trace reflects the time course of the amount of vesicles resting in the alkaline state. Nevertheless, the number of alkaline vesicles depends on the number of exocytosed vesicles. Therefore, we set out to measure the maximal relative size of the alkaline vesicle population, which is determined by the maximal number of alkaline vesicles detected in the acid phase in relation to the maximal number of vesicles being exocytosed. To estimate the maximal alkaline vesicle population size with respect to the recycling pool, corresponding amplitudes of fluorescence measured at maximum under pH 5.5 and pH 7.5 were divided by each other (Figure 3 (C)). As depicted in Figure 3 (D), the relative size of the alkaline vesicle population significantly expanded with increasing numbers of action potentials (p<0.005). Notably, the relative size of the alkaline vesicle population was larger at stimulus strengths that recruit at least 60% of the recycling pool [28].

In conclusion, here we present that the relative size of the alkaline vesicle population significantly depends on the number of action potentials, rising with increasing number of action potentials.

### The relative size of the alkaline vesicle population depends on the frequency of action potentials

After analyzing the influence of the number of action potentials on the relative size of the alkaline vesicle population, the impact of the stimulus frequency was investigated. Therefore, the stimulus frequency was changed in the following experiments while keeping the action potential number constant at 200 pulses. Again, pH-cycling was performed and neurons were stimulated electrically with 200 action potentials under changing frequency (10 Hz, 20 Hz, 40 Hz and 80 Hz) (N = 5, n = 163).

**Figure 4:**
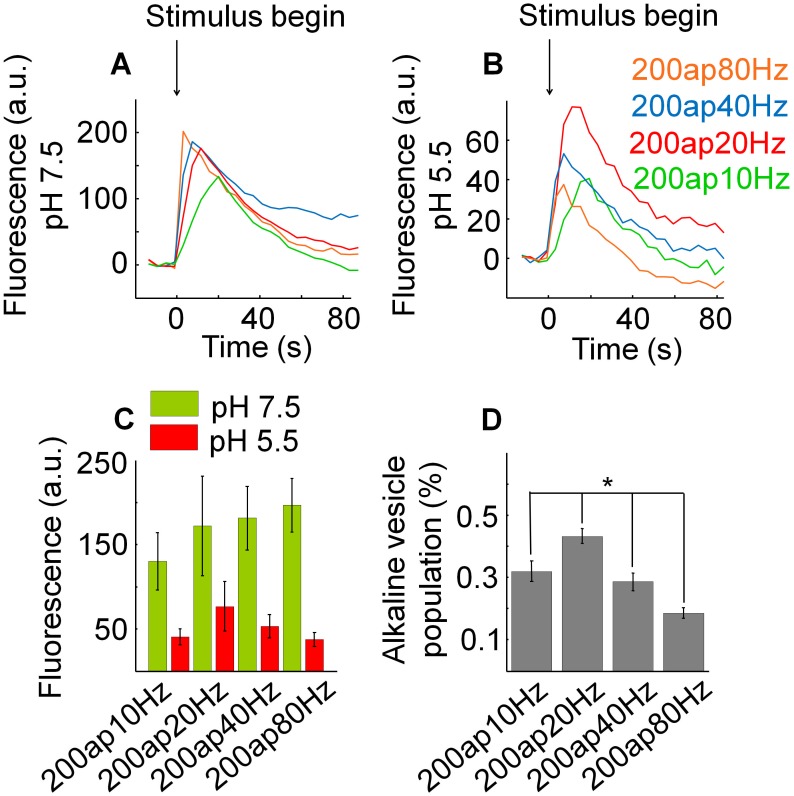
The relative size of the alkaline vesicle population depends on the stimulus frequency. (A) Mean fluorescence profiles at pH 7.5 for different action potential frequencies with 200 pulses each: 10 Hz, 20 Hz, 40 Hz, 80 Hz (N = 5, n = 163). Increasing the stimulus frequency caused a non-significant increase of fluorescence (p = 0.248). (B) Mean fluorescence profiles at pH 5.5 for different action potential frequencies with 200 pulses each: 10 Hz, 20 Hz, 40 Hz, 80 Hz (N = 5, n = 163). Increasing the stimulus frequency led to an decrease of fluorescence for frequencies above 10 Hz which was not significant either (p = 0.525). (C) Mean fluorescence changes at pH 5.5 and 7.5 induced by different action potential frequencies with 200 pulses each: 10 Hz, 20 Hz, 40 Hz, 80 Hz (N = 5, n = 163). To estimate the maximal alkaline vesicle population size with respect to the recycling pool, corresponding amplitudes of fluorescence measured at maximum under pH 5.5 and pH 7.5 were divided by each other. (D) Relative size of the alkaline vesicle population depending on the action potential frequency (N = 5, n = 163). The relative size of the alkaline vesicle population significantly depends on the stimulus frequency (p = 0.015).

At the images taken at pH 7.5 increasing the action potential frequency induced an increase of the amplitude of the fluorescence trace at maximum (Figure 4 (A)). Nonetheless, this effect turned out to be insignificant (p = 0.248). At the images taken at pH 5.5 increasing the action potential frequency led to a decrease of the amplitude of the fluorescence trace at maximum for frequencies above 10 Hz, which was also insignificant (p = 0.525) (Figure 4 (B)). Again the ratio of the maximal alkaline vesicle population size with respect to the recycling pool was calculated (Figure 4 (C)) and an inverse relationship between this ratio and the stimulus frequency was found: As shown in Figure 4 (D), with increasing action potential frequency, the number of alkaline vesicles in relation to the number of exocytosed vesicles decreased significantly for frequencies above 10 Hz (p = 0.015).

In summary, here we show that the relative size of the alkaline vesicle population significantly depends on number and frequency of action potentials, rising with increasing action potential number and decreasing frequency. Notably, our measurements revealed a maximal relative size of the alkaline pool of 40% under moderate stimulation with 200ap20Hz, a physiological stimulus that is assumed to partly release the recycling pool [27], [28]. These results are in line with previous work which showed that the alkaline vesicle population had a relative size of 30% or 37% compared to 100% of exocytosed vesicles, respectively [6], [8].

### Variation of the relative size of the alkaline vesicle population can be predicted by modeling

Now we were interested in whether variation of the relative size of the alkaline vesicle population could be predicted by modeling. Therefore, we developed two different models based on an established model for vesicle population dynamics [6], [7]. The “ideal” model simply depicts the dynamics of different vesicle populations upon and after stimulation. The “realistic” model also takes into account the processes during pH-cycling. It contains simulation of the pH-phases and considers the fact that vesicles endocytosed upon external pH 5.5 are already acidified [6] and do not contribute to the alkaline pool. With these two models, we simulated the dynamics of the surface synapto-pHluorin pool and the alkaline vesicle population at different stimulus conditions. The time constants for endocytosis (τ endo  = 17 seconds) and reacidification (τ reac  = 4 seconds) derived from current literature [2] and have been shown to be valid for a range of moderate stimuli. They were modeled to be invariable with regard to the stimulus condition.

The results yielded by both models were qualitatively similar. According to the “ideal” model, the relative size of the alkaline vesicle population (in relation to the surface pool) increased for increasing action potential number (Figure S5 (A)) although less pronounced than in the experimental data. The relative peak size of the alkaline vesicle population decreased for increasing stimulus frequency (Figure S5 (B)), but the decrease was less marked than in the experiments. In the experimental data, the relative alkaline vesicle population upon 400ap20Hz in the action potential number series and upon 200ap10Hz in the action potential frequency series did not match into the trend. In the case of 400ap20Hz, the reason might be the following: While the endocytosis rate might be constant for a range of moderate stimuli, it has been shown to slow down for intense stimuli [2], [18], [31]. According to our model, slower endocytosis (τ endo  = 19 seconds) resulted in a smaller relative alkaline vesicle population size upon 400ap20Hz.

According to the “realistic” model, the relative size of the alkaline vesicle population upon all stimulation paradigms turned out to be lower than according to the “ideal” model (Figure S5 (C+D)). This is readily explained by the fact that vesicles endocytosed upon external pH 5.5 are already acidified and do not contribute to the alkaline vesicle population [6].

For better demonstration, the surface pool and the alkaline vesicle population simulated with the “ideal” model (Figure S5 (E)) and with the “realistic” model (Figure S5 (F)) are depicted. As stimulation paradigms 100ap20Hz were supposed.

In conclusion, both models, whether they consider the effect of pH-cycling on the vesicle dynamics or not, show similar results. They mirror the experimentally obtained characteristics of the relative alkaline vesicle population upon different stimulus conditions. However, according to both models, the simulated data revealed a smaller alkaline vesicle population than measured in our experiments. In these simulations, we assumed that the time constants of endocytosis and reacidification are invariable with regard to the stimulation paradigms. In reality, these parameters might change along with the stimulus conditions and introduce further variations. Therefore, we set out to test our hypotheses and tried to get access to both endocytosis and reacidification time constants in the following.

### The reacidification time constant depends both on action potential number and frequency

As a next step, reacidification kinetics was experimentally identified as a decaying exponential function with a timeconstant τ reac for all stimulation paradigms according to the protocol shown in Figure 1(A). In our pH-cycling experiments at neurons transfected with synapto-pHluorin, the reacidification time constant ranged between 9 and 23 seconds, decreasing with rising action potential number and increasing with rising stimulus frequency (Tables 1 and 2). Our results are unfortunately contrary to previous work which found a shorter reacidification time constant of 4–5 seconds that was invariable with regard to the stimulus condition [6].

### Calculating the endocytosis time constant upon changing stimulation paradigms by deconvolution

**Figure 5:**
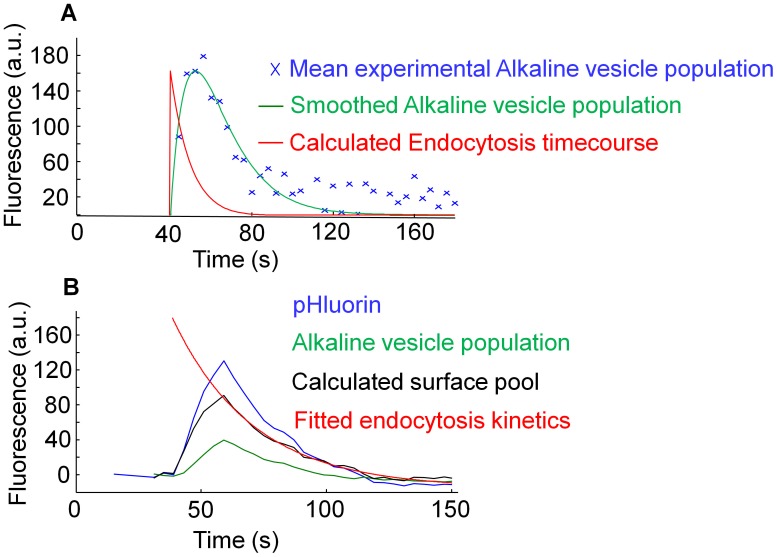
Two different approaches for the calculation of the endocytosis time constant. (A) Deconvolution of the reacidification time course from the alkaline vesicle population dynamics and estimated endocytosis time course. For analysis, experimental data from the action potential number series was used (stimulation paradigms: 200 pulses at 20 Hz). Stimulus begin was at 40 seconds. (B) Calculation of the time course of the surface pool, which is determined by the alkaline vesicle population (fluorescence time course at pH 5.5) subtracted from the total pHluorin (fluorescence time course at pH 7.5). The endocytosis time constants were calculated by monoexponential curve fitting. Stimulus begin was at 40 seconds.

Assuming that the alkaline vesicle population size depends only on the amount of endocytosed vesicles (input) and on the amount of acidified vesicles (output), the hitherto existing data implicate that the amount of endocytosed vesicles can be appraised by determination of the alkaline vesicle population size. Therefore, in the following we aimed to estimate the endocytosis time course by deconvolution of the reacidification time course from the alkaline vesicle population dynamics. This allows for the calculation of the time course of endocytosis for various stimuli. Then, deconvolution was performed as described in current literature [6] (Figure 5 (A)), and the time constants for endocytosis of the mean fluorescence time courses upon different stimulation paradigms were calculated (Tables 3 and 4, for further details see also the materials and methods section). Unfortunately, it was not possible to calculate an endocytosis time constant for 50ap20Hz due to the low signal-to-noise ratio. We found that the time constant for endocytosis increased with rising action potential number and decreasing stimulus frequency. These results are in line with earlier work, as it has been shown that the endocytosis time constant ranges between 4 and 90 seconds and depends both on action potential number and frequency [25].

**Table 3:** The time constant for endocytosis derived by deconvolution depends on the action potential number.

Action potential number	τ endo (s)
50ap20Hz	Not possible to calculate due to low signal-to-noise ratio
100ap20Hz	8.40
200ap20Hz	11.06
400ap20hz	28.56

**Table 4:** The time constant for endocytosis derived by deconvolution shows no clear dependency on the stimulus frequency.

Action potential frequency	τ endo (s)
200ap10Hz	20.37
200ap20Hz	13.76
200ap40Hz	10.67
200ap80hz	7.13

### Calculating the endocytosis time constant upon changing stimulation paradigms by monoexponential curve fitting on the decaying surface pool time course

Performing pH-cycling, we show the first dynamic measurements of the alkaline vesicle population. Consequently, we are able to calculate the time course of the surface pool, which is determined by the alkaline vesicle population (fluorescence time course at pH 5.5) subtracted from the total pHluorin (fluorescence time course at pH 7.5). Doing this, the decay of the surface pool time course reflects the kinetics of endocytosis, enabling us to calculate the time constant for endocytosis by monoexponential curve fitting (Figure 5 (B)). This alternative way to get access to endocytosis time constants is useful as deconvolution is prone to some problems: First, it is very noise-sensitive, which is not ideal in biological systems. And second, for deconvolution, further assumptions have to be made, for example the assumption that the kinetics of endocytosis is time invariant. However, if there was indeed a readily retrievable pool [22], this assumption would not be fulfilled. Therefore, we set out to calculate the time course of the surface pool to access the endocytosis time constants as depicted in Figure 5 (B). The resulting endocytosis time constants (Tables 5 and 6) showed no clear dependency and were longer than the ones derived by deconvolution.

**Table 5:** The time constant for endocytosis calculated by the decay of the surface pool shows no clear dependency on the action potential number.

Action potential number	τ endo (s)
50ap20Hz	35.43
100ap20Hz	52.06
200ap20Hz	27.77
400ap20hz	58.48

**Table 6:** The time constant for endocytosis calculated by the decay of the surface pool shows no clear dependency on the stimulus frequency.

Action potential frequency	τ endo (s)
200ap10Hz	35.34
200ap20Hz	22.67
200ap40Hz	31.32
200ap80hz	29.86

### Modeling the relative size of the alkaline vesicle population based on stimulation-dependent endocytosis and reacidification time constants

**Figure 6:**
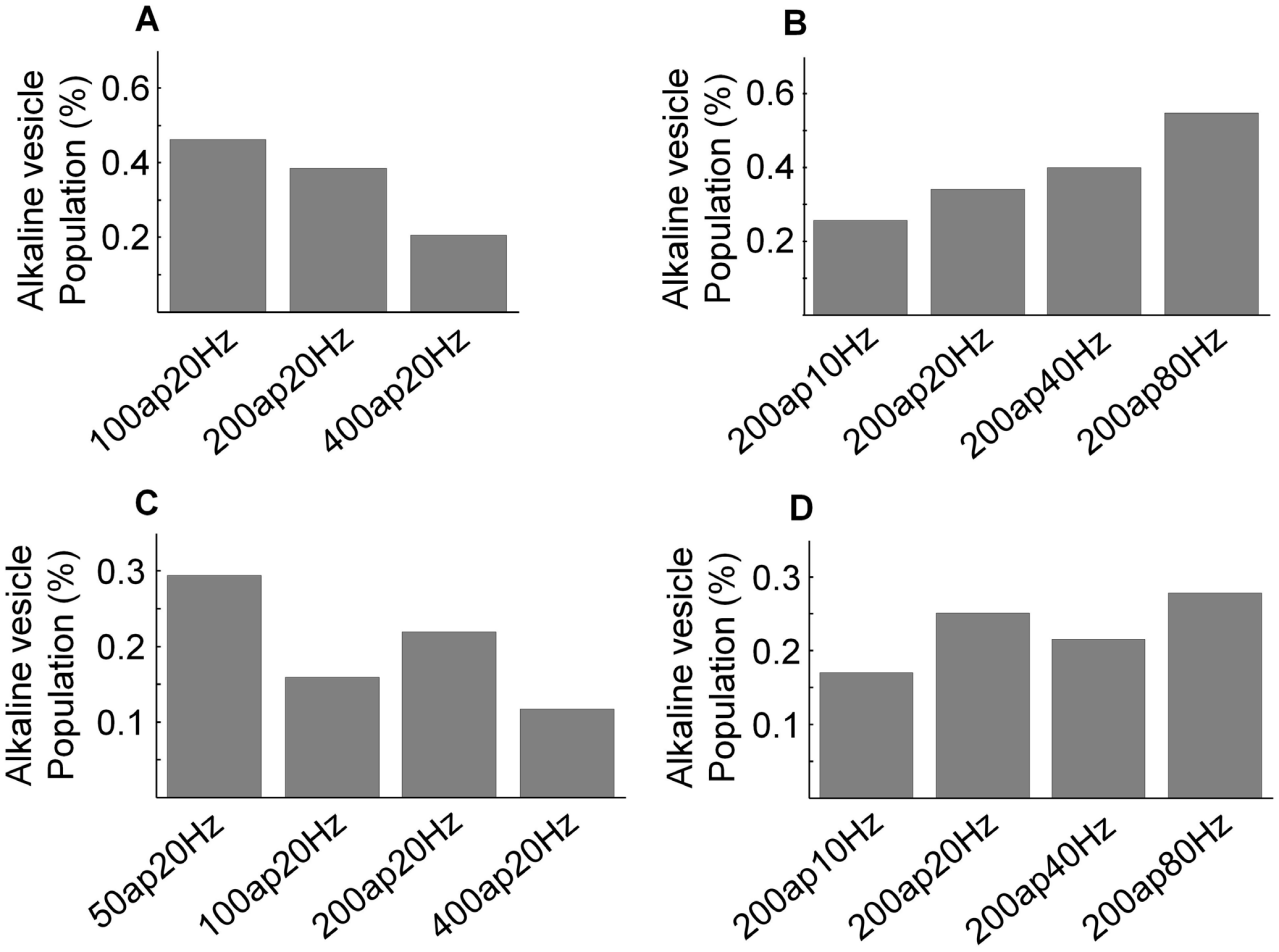
Variation of the relative size of the alkaline vesicle population can be predicted by modeling. For simulation, the time constants for endocytosis and for reacidification were modeled to be variable to the stimulus condition. (A) Relative size of the alkaline vesicle population depending on the action potential number according to the “ideal” model. Endocytosis time constants were derived by deconvolution. The relative alkaline vesicle population size decreased with rising number of action potentials. The stimulus 50ap20Hz was omitted, as it was not possible to calculate an endocytosis time constant by deconvolution due to low signal-to-noise ratio. (B) Relative size of the alkaline vesicle population depending on the action potential frequency according to the “ideal” model. Endocytosis time constants were derived by deconvolution. The relative alkaline vesicle population size increased with rising number of action potential frequency. (C) Relative size of the alkaline vesicle population depending on the action potential number according to the “ideal” model. Endocytosis time constants were derived by monoexponential curve fitting from the decaying surface pool time course. The calculated relative alkaline vesicle population size reflects the experimentally defined alkaline vesicle population dynamics (Figure 3) except for stimulation with 50ap20Hz. (D) Relative size of the alkaline vesicle population depending on the action potential frequency according to the “ideal” model. Endocytosis time constants were derived by monoexponential curve fitting from the decaying surface pool time course. The calculated relative alkaline vesicle population size reflects the experimentally defined alkaline vesicle population dynamics (Figure 4) except for stimulation with 200ap80Hz.

After having shown that both endocytosis and reacidification time constants were variable with regard to the stimulus condition, we changed our hypotheses for simulation of the alkaline vesicle population accordingly. Based on the newly derived time constants (experimentally defined reacidification time constants and mathematically defined endocytosis time constants, on the one hand obtained by the surface pool-method, on the other hand obtained by deconvolution) we now set out to perform the simulation once again. In both cases the mathematically derived data according to the “ideal” model did not fit well to the experimental data (Figure 6), however, when applying the surface pool-method to calculate the endocytosis time constants, the simulation fits better (Figure 6 (C+D)).

In conclusion, our data indicate that the dynamics of the alkaline vesicle population can be predicted by modeling. However, the simulation strongly depends on the hypotheses that are made, and unknown variables lead to mathematical uncertainty. Our simulation data in summary prove that it is typically a challenging endeavor to predict a biological system by a mathematical model which is based on simplifying assumptions.

### Simulation: The effect of pH-cycling can be predicted by modeling

Performing pH-cycling, only those synaptic vesicles that have been endocytosed upon external pH of 7.5 will be detected during the acidic period due to their alkaline and thus fluorescent state. In addition to the “ideal” simulation, which reflects the real dynamics of the alkaline vesicle population, the “realistic” simulation also takes the changing external pH phases into account and consequently mirrors the underestimation of the relative size of the alkaline vesicle population. Therefore, in the following we were interested in analyzing the extent of error made by measuring the alkaline vesicle population size upon pH-cycling. For analysis, a “realistic” model with balanced phases lasting 2 seconds was simulated. To simplify mathematical work, in our model the reported time constants for endocytosis (τ endo  = 17 seconds) and reacidification (τ reac  = 4 seconds) were assumed [2].

As depicted in Figure S6 (A), pH-cycling leads to an underestimation of the relative size of the alkaline vesicle population. Nevertheless, this underestimation ranges in a narrow frame between 30% and 40% depending on the stimulation paradigms and no clear trend can be distinguished. Consequently, we conclude that the findings generated by our pH-cycling experiments are reliable data.

Furthermore, additionally to our data derived from the experiments with unbalanced perfusion phase length we set out to analyze the influence of the duration of the two phases on the number of detected alkaline vesicles. Again, we simulated a “realistic” model that considered the changing perfusion conditions. Figure S6 (B) indicates that the underestimation of the relative size of the alkaline vesicle population measured upon pH-cycling clearly depends on the perfusion interval length: Upon pH-cycling with 4 seconds pH 5.5 and 2 seconds pH 7.5 the relative alkaline vesicle population size is more underestimated than upon pH-cycling with 2 seconds pH 5.5 and 4 seconds pH 7.5. This is in line with our experimental data (Figure S4).

## Discussion

In the present paper, we probed the feasibility of time resolved measurements of the alkaline vesicle population of synaptic vesicles by fast cycling of external pH. We were able to demonstrate that neuronal alkaline vesicles are mainly synaptic and can be detected with different pHluorins (synapto-pHluorin and VGLUT1-pHluorin). We show that the high amount of surface fluorescence of synapto-pHluorin (Figure 1 (A)) is essential for the analytical separation of images obtained at different external pH applications (Figure 2, Figure S2). Furthermore, our data indicate that the maximal amount of alkaline vesicles reached upon stimulation increases with rising number of action potentials (Figure 3). This is predicted by a simple mathematical model based on published values (Figure S5 (A+C)). Interestingly, we found that for a high amount of action potentials (400 pulses at 20 Hz) the fraction of alkaline vesicles was lower than expected. This could be explained by this kind of stimulus already provoking bulk endocytosis at hippocampal terminals [31].

Besides the influence of the action potential number on the relative alkaline vesicle population, we were able to show that the alkaline vesicle population decreases with increasing stimulation frequency except for stimulation with 200ap10Hz (Figure 4) and that this finding is again predicted by the simple model (Figure S5 ((B+D)).

However, the interpretation of alkaline vesicle fluorescence measured upon pH-cycling has to be done with care. We could demonstrate that synaptic vesicle recycling is reversibly impaired at low external pH (Figure S3). The amount of alkaline vesicles detected thus depends on time-point and length of pH 5.5 application. Moreover, vesicles endocytosed upon external pH 5.5 are already acidic and do not contribute to the alkaline vesicle fluorescence so that the size of the pool is underestimated when cycling with acidic pH. We were able to demonstrate this experimentally (Figure S4) and mathematically, simulating the alkaline vesicle population upon changing perfusion conditions (Figure S6 (B)). On the other hand, the amount of underestimation upon pH-cycling with balanced phase lengths varies only slightly with the stimulus paradigms (Figure S6 (A)). According to the simulation, the modeled alkaline vesicle population still increases with the number of action potentials and decreases with rising stimulus frequency so that the results from our simulation fit to the experimental data. Notably, the impact of number of action potentials and stimulation frequency were far more pronounced in our experimental data than as predicted by the simulation. A possible explanation for this may be that we assumed the time constants for endocytosis and reacidification to be constant for different stimuli. It is however likely that they vary considerably and that this may account for the observed differences. Particularly, changes in the reacidification time constant would influence the results of the simulation. It is likely that reacidification, which is an energy dependent process, is dependent on the availability of energy carriers which varies with stimulation intensity. Therefore, we experimentally defined the reacidification time constant upon changing stimulation paradigms and found indeed a stimulation-dependent variability, ranging between 9 and 23 seconds (Tables 1 and 2). This is in contrast to previous work [2], [6]. However, our data unambiguously showed a linear dependency of the reacidification time on the stimulus condition, and previous work did not systematically analyze the stimulation dependency. Based on these values, we calculated the endocytosis time constants upon changing stimulation paradigms using two different approaches: On the one hand, we estimated the endocytosis time course by deconvolution of the reacidification time course from the alkaline vesicle population dynamics and found a dependency of the endocytosis time constant on the action potential number, as it was reported earlier [25]. On the other hand, as we have performed dynamic measurements of the alkaline vesicle population, we had the opportunity to calculate the endocytosis time constant by monoexponential curve fitting on the decaying surface pool time course. The resulting data showed longer time constants and no clear linear dependency on the stimulus condition. The differences in the endocytosis time constants in our experimental data probably occur due to differences in the analytical approach, however, calculation by deconvolution is more susceptible to mistakes as it is based on simplifying assumptions, and a high time resolution is needed, which cannot be provided for pH-cycling to date. In literature, endocytosis time constants between 4 and 90 seconds depending on the stimulation paradigms were reported [25]. Another previous work found an endocytosis time constant of 15 seconds after a single nerve impulse or short burst stimulation [2], thus under those stimulus conditions, when a very fast mode of endocytosis, kiss-and-run, with retrieval in 1 second or less was reported [32]. Heterogenous stimulation paradigms and differences in the analytical approach may account for the diverging data found in literature, but these data indicate that there is a wide range in the kinetics of endocytic retrieval.

When we changed our hypotheses for simulation and assumed both endocytosis and reacidification time constants to be variable with regard to the stimulus condition, the modeling did not fit well to the experimental data in both cases. However, when applying the surface pool-method to calculate the endocytosis time constants, the simulation fits better (Figure 6). Notably, the short stimuli (50ap20Hz and 200ap80hz) did not match into the trend. This may be due to the fact that the cycling effect is more crucial if the stimulus is very short and therefore it is very important that the position of the perfusion system is as exact as possible. Another reason could be that the assumptions, on which the mathematical model is based, do not fit well for those short stimuli.

In conclusion, our data indicate that the simulated results strongly depend on the hypotheses which are made for simulation, and variations in the reacidification time constants of just one second lead to substantial different results in our simulated data. However, the living cell is not a static system, but may be influenced by a lot of parameters, which may not be totally considered in a mathematical model based on simplifying assumptions. Consequently, for the interpretation of simulated data based on pH-cycling experiments the following limitations have to be considered: First, there are at least two unknown variables, the time constant for endocytosis and the time constant for reacidification, which lead to mathematical uncertainty. Second, the pH-cycling itself complicates the simulation, leading to an underestimation of the alkaline vesicle fraction. And third, the time resolution of 4 seconds is the best we could do performing pH-cycling, but is slow for the calculation of endocytosis time constants by deconvolution. Consequently, for the calculation of endocytosis time constants, the surface pool-method should be preferred as it is more robust.

We nonetheless are of the opinion that our mathematical model greatly adds to our work about the alkaline vesicle population, providing the opportunity for further analysis of the alkaline vesicle population. For interpretation, the hypotheses concerning the unknown variables as well as the limited time resolution have to be considered and further analyzed.

Taking the results of our study into account, we suppose that short electrical stimuli deplete the alkaline vesicle population whereas under long lasting stimulation, the alkaline vesicle population increases. We show that the dynamics of the alkaline vesicle population can directly be accessed performing pH-cycling and that the alkaline vesicle population size reflects the amount of endocytosed vesicles.

In summary, this study provides an extensively probed methodical framework for dynamic measurements of the alkaline population of synaptic vesicles that paves the way to the direct assessment of endocytosis time constants. Our data indicate that analyzing the dynamics of the alkaline vesicle population may provide new insights into the kinetics of endocytic retrieval.

## Supporting Information

Figure S1 **pH-cycling protocol.** At the beginning, an acid-pulse was performed to test the position of the perfusion system. Then, neurons were perfused with saline of pH 7.5 to allow baseline to recover. At 28 seconds, pH-cycling was started. Electrical stimulation started at 40 seconds.
(TIF)

Figure S2 **Determination of the alkaline vesicle population with VGLUT1-pHluorin.** (A) Mean fluorescence time course of VGlut1-pHluorin before separation of the images (n = 7). Inset: Exemplary mean fluorescence time course of VGLUT1-pHluorin of a single experiment before separation of the images. (B) Magnification of the red rectangle marked in (A). Note that the surface fluorescence reaches a steady-state level upon pH 7.5. (C) Representative difference images before and after stimulation of neurons transfected with VGLUT1-pHluorin. (D) Mean fluorescence profiles at pH 7.5 for 200 (N = 7, n = 115) and 100 action potentials (N = 7, n = 96) at 20 Hz each. (E) Mean fluorescence profiles at pH 5.5 for 200 (N = 7, n = 115) and 100 action potentials (N = 7, n = 96) at 20 Hz each. (F) Relative size of the alkaline vesicle population depending on the action potential number measured with VGLUT1-pHluorin (N = 7) and synapto-pHluorin (N = 6). In contrast to synapto-pHluorin (p = 0.040), there was no significant difference in the relative size of the alkaline vesicle population depending on the action potential number measured with VGLUT1-pHluorin (p = 0.374). (G) Normalized relative size of the alkaline vesicle population depending on the action potential number measured with VGLUT1-pHluorin (N = 7) and synapto-pHluorin (N = 6). The relative part of the alkaline vesicle population upon stimulation with 200ap20Hz is valued to 1. There was no significant difference in the relative alkaline vesicle population size upon stimulation with 100ap20Hz when measured with synapto-pHluorin or VGLUT1-pHluorin (p = 0.780).
(TIF)

Figure S3 **Low external pH reversibly reduces the amount of endocytosed synaptic vesicles.** (A) Exemplary fluorescence time course at pH 7.5. Neurons were loaded with the styryl dye FM 1–43. At 30 seconds, unloading was performed by stimulation with 200ap20Hz. (B) Representative images of neurons loaded with the styryl dye FM 1–43 at pH 7.5 (left) and at pH 5.5 (right). (C) Mean total fluorescence at pH 7.5 and pH 5.5 after loading with the styryl dye FM 1–43. Upon pH 7.5, higher amount of FM 1–43 was loaded than upon pH 5.5. However, this effect was not significant (pH 7.5 (N = 6): mean total fluorescence  = 1029 a.u.±155.61 SEM; pH 5.5 (N = 4): mean total fluorescence  = 581.52 a.u.±164.76 SEM, p = 0.092). (D) Mean dF exocytosis at pH 7.5 and pH 5.5 upon stimulation with 200ap20Hz. The difference in fluorescence decrease due to unloading of FM 1–43 turned out to be significant (pH 7.5 (N = 6): 31.33%±2.15 SEM; pH 5.5 (N = 3): 19.6%±2.47 SEM, p = 0.013). (E) Normalized mean fluorescence profiles at pH 7.5 (N = 7, n = 5286) and pH 5.5 (N = 3, n = 2607) upon stimulation with 200ap20Hz.
(TIF)

Figure S4 **The relative size of the alkaline vesicle population depends on the perfusion interval length.** (A) Representative mean fluorescence profile upon pH-cycling with doubled perfusion interval length of pH 7.5. (B) Magnification of the red rectangle marked in (A). (C) Representative mean fluorescence profile upon pH-cycling with doubled perfusion interval length of pH 5.5. (D) Magnification of the red rectangle marked in (C). (E) The relative size of the alkaline vesicle population significantly depends on the perfusion interval length (2 s acid solution +4 s alkaline solution (N = 6, n = 274): 14.55%±0.05 SEM; 2 s alkaline solution +4 s acid solution (N = 6, n = 274): 46.26%±0.11 SEM, p = 0.025).
(TIF)

Figure S5 **Variation of the relative size of the alkaline vesicle population can be predicted by modeling. For simulation, the time constants for endocytosis and for reacidification were modeled to be invariable to the stimulus condition.** (A) Relative size of the alkaline vesicle population depending on the action potential number according to the “ideal” model. The relative alkaline vesicle population size increased with rising number of action potentials. (B) Relative size of the alkaline vesicle population depending on the action potential frequency according to the “ideal” model. The relative alkaline vesicle population size decreased with rising number of action potential frequency. (C) Relative size of the alkaline vesicle population depending on the action potential number according to the “realistic” model. The relative alkaline vesicle population size increased with rising number of action potentials, but was generally smaller compared to the data obtained by the “ideal” model due to underestimation. (D) Relative size of the alkaline vesicle population depending on the action potential frequency according to the “realistic” model. The relative alkaline vesicle population size decreased with rising action potential frequency, but was generally smaller compared to the data obtained by the “ideal” model due to underestimation. (E) Simulated time courses of the surface pool and the alkaline vesicle population according to the “ideal” model. As stimulation paradigms 100 pulses at 20 Hz were assumed. (F) Simulated time courses of the surface pool and the alkaline vesicle population according to the “realistic” model. As stimulation paradigms 100 pulses at 20 Hz were assumed. Vesicles endocytosed upon external pH 5.5 are already acidified and do not contribute to the alkaline pool.
(TIF)

Figure S6 **Underestimation effects upon pH-cycling.** (A) Underestimation effect upon pH-cycling with balanced perfusion intervals (2 s pH 5.5, 2 s pH 7.5) depending on action potential number and action potential frequency according to the “realistic” model. (B) Underestimation effect upon pH-cycling depending on the acid and alkaline phase length according to the “realistic” model.
(TIF)

Methods S1 **Simulation of the alkaline vesicle population.**
(DOCX)

Text S1 **Performing pH-cycling with VGLUT1-pHluorin.**
(DOCX)
