# Supplementary figures and images for: Dynamic Properties of the Alkaline Vesicle Population at Hippocampal Synapses
Source: PLoS One. 2014 Jul 31;9(7):e102723. doi: 10.1371/journal.pone.0102723 (PMC4117485; doi:10.1371/journal.pone.0102723)

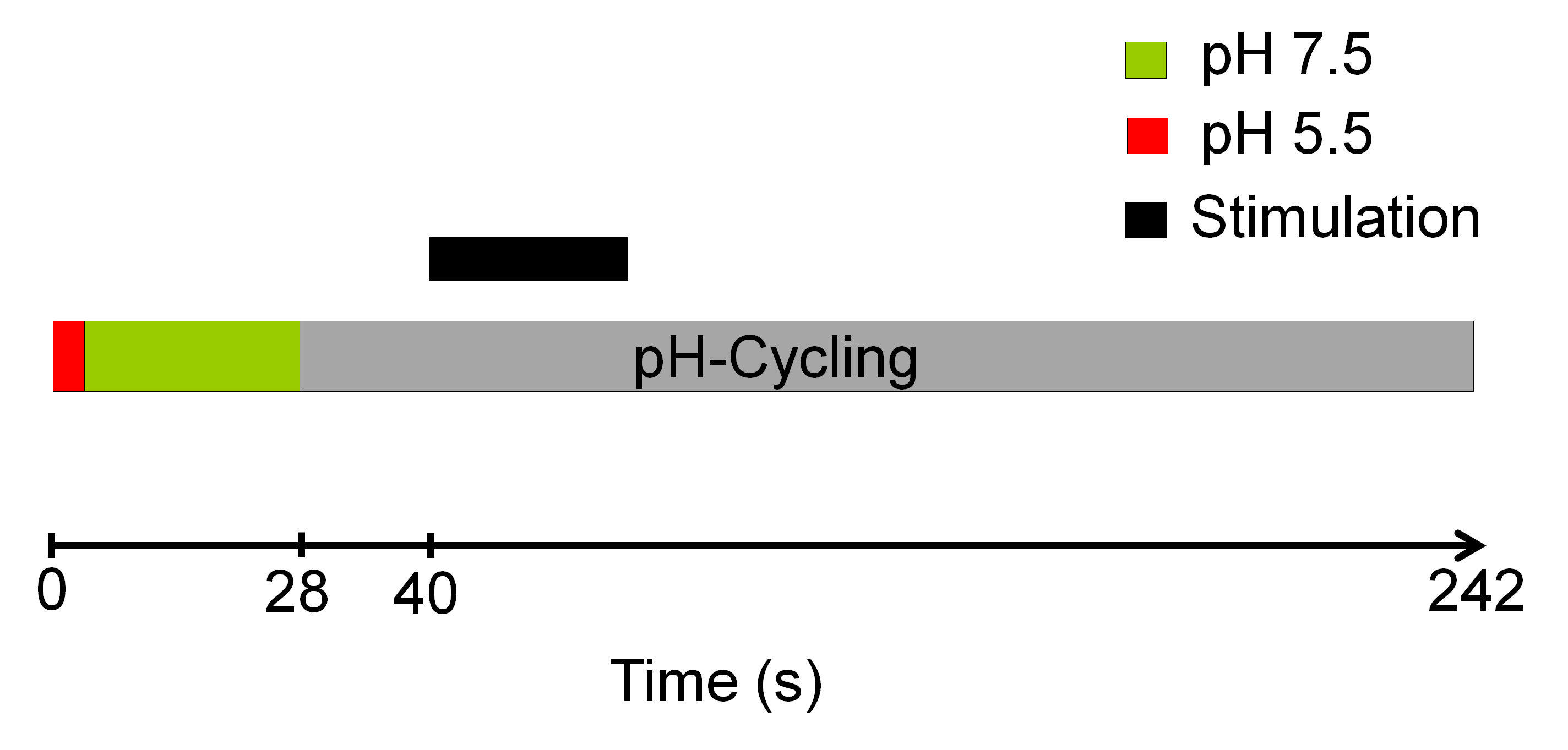

Supplement: Figure S1 — pH-cycling protocol. At the beginning, an acid-pulse was performed to test the position of the perfusion system. Then, neurons were perfused with saline of pH 7.5 to allow baseline to recover. At 28 seconds, pH-cycling was started. Electrical stimulation started at 40 seconds. (TIF) [file pone.0102723.s001.tif]

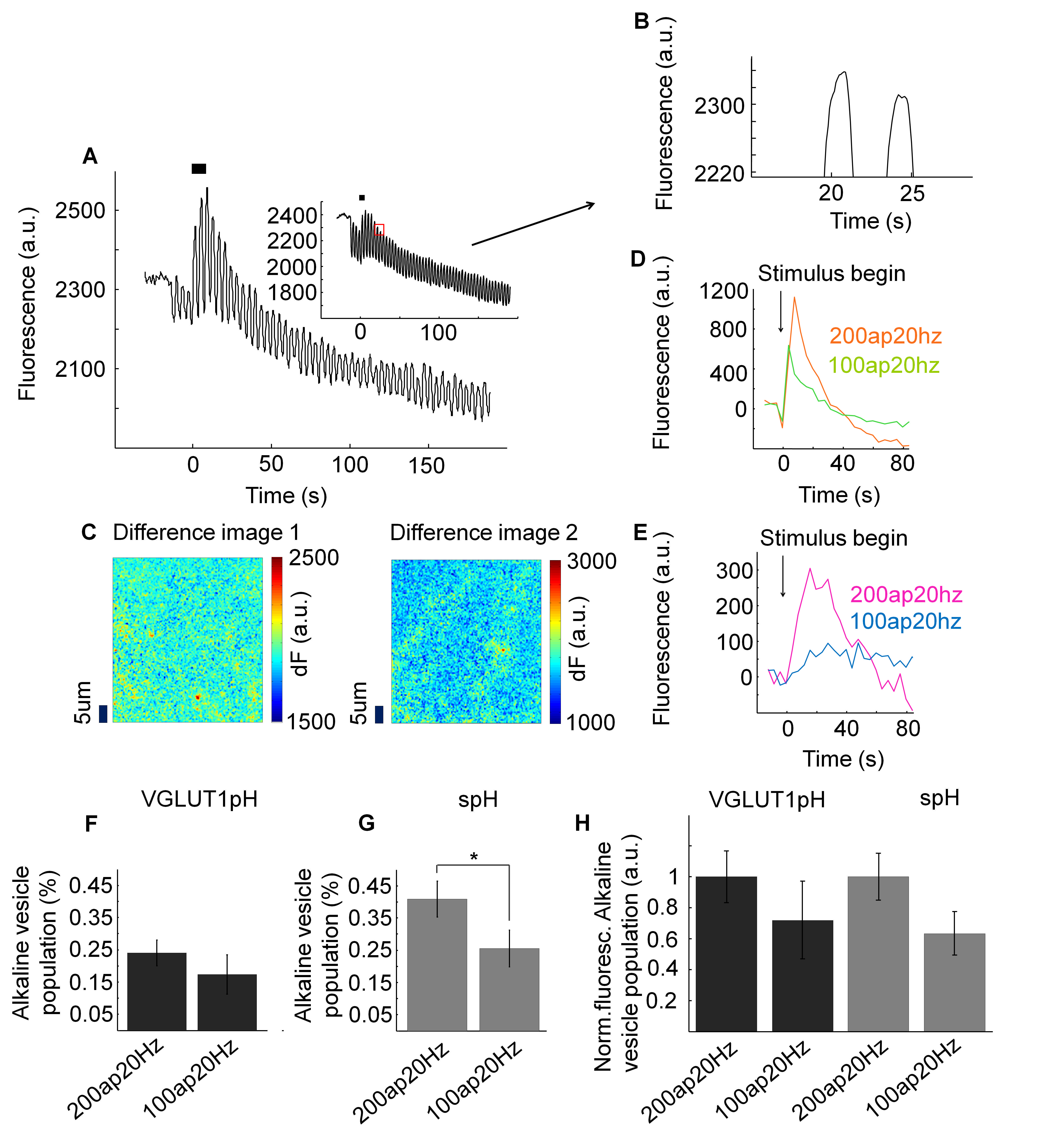

Supplement: Figure S2 — Determination of the alkaline vesicle population with VGLUT1-pHluorin. (A) Mean fluorescence time course of VGlut1-pHluorin before separation of the images (n = 7). Inset: Exemplary mean fluorescence time course of VGLUT1-pHluorin of a single experiment before separation of the images. (B) Magnification of the red rectangle marked in (A). Note that the surface fluorescence reaches a steady-state level upon pH 7.5. (C) Representative difference images before and after stimulation of neurons transfected with VGLUT1-pHluorin. (D) Mean fluorescence profiles at pH 7.5 for 200 (N = 7, n = 115) and 100 action potentials (N = 7, n = 96) at 20 Hz each. (E) Mean fluorescence profiles at pH 5.5 for 200 (N = 7, n = 115) and 100 action potentials (N = 7, n = 96) at 20 Hz each. (F) Relative size of the alkaline vesicle population depending on the action potential number measured with VGLUT1-pHluorin (N = 7) and synapto-pHluorin (N = 6). In contrast to synapto-pHluorin (p = 0.040), there was no significant difference in the relative size of the alkaline vesicle population depending on the action potential number measured with VGLUT1-pHluorin (p = 0.374). (G) Normalized relative size of the alkaline vesicle population depending on the action potential number measured with VGLUT1-pHluorin (N = 7) and synapto-pHluorin (N = 6). The relative part of the alkaline vesicle population upon stimulation with 200ap20Hz is valued to 1. There was no significant difference in the relative alkaline vesicle population size upon stimulation with 100ap20Hz when measured with synapto-pHluorin or VGLUT1-pHluorin (p = 0.780). (TIF) [file pone.0102723.s002.tif]

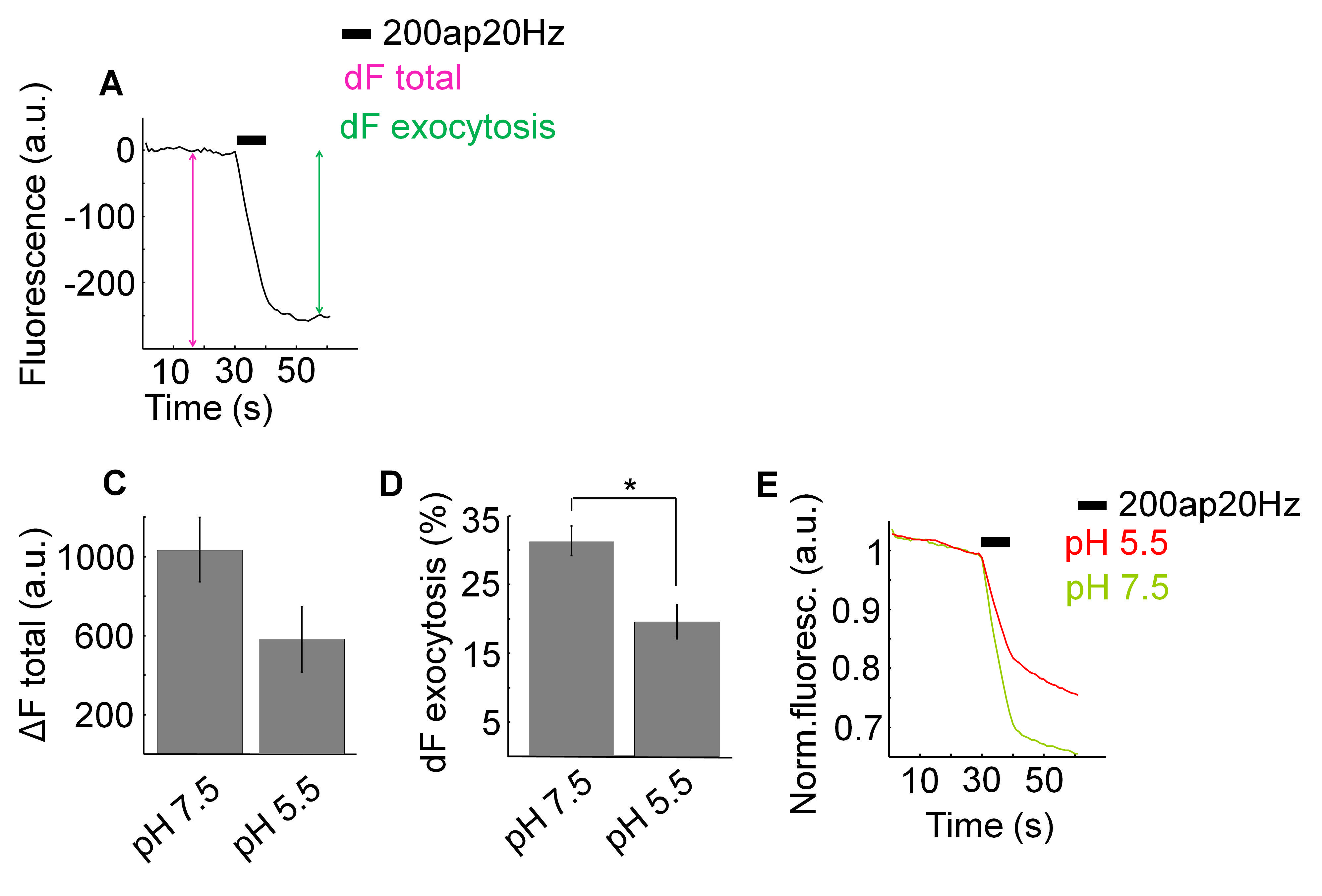

Supplement: Figure S3 — Low external pH reversibly reduces the amount of endocytosed synaptic vesicles. (A) Exemplary fluorescence time course at pH 7.5. Neurons were loaded with the styryl dye FM 1–43. At 30 seconds, unloading was performed by stimulation with 200ap20Hz. (B) Representative images of neurons loaded with the styryl dye FM 1–43 at pH 7.5 (left) and at pH 5.5 (right). (C) Mean total fluorescence at pH 7.5 and pH 5.5 after loading with the styryl dye FM 1–43. Upon pH 7.5, higher amount of FM 1–43 was loaded than upon pH 5.5. However, this effect was not significant (pH 7.5 (N = 6): mean total fluorescence = 1029 a.u.±155.61 SEM; pH 5.5 (N = 4): mean total fluorescence = 581.52 a.u.±164.76 SEM, p = 0.092). (D) Mean dF exocytosis at pH 7.5 and pH 5.5 upon stimulation with 200ap20Hz. The difference in fluorescence decrease due to unloading of FM 1–43 turned out to be significant (pH 7.5 (N = 6): 31.33%±2.15 SEM; pH 5.5 (N = 3): 19.6%±2.47 SEM, p = 0.013). (E) Normalized mean fluorescence profiles at pH 7.5 (N = 7, n = 5286) and pH 5.5 (N = 3, n = 2607) upon stimulation with 200ap20Hz. (TIF) [file pone.0102723.s003.tif]

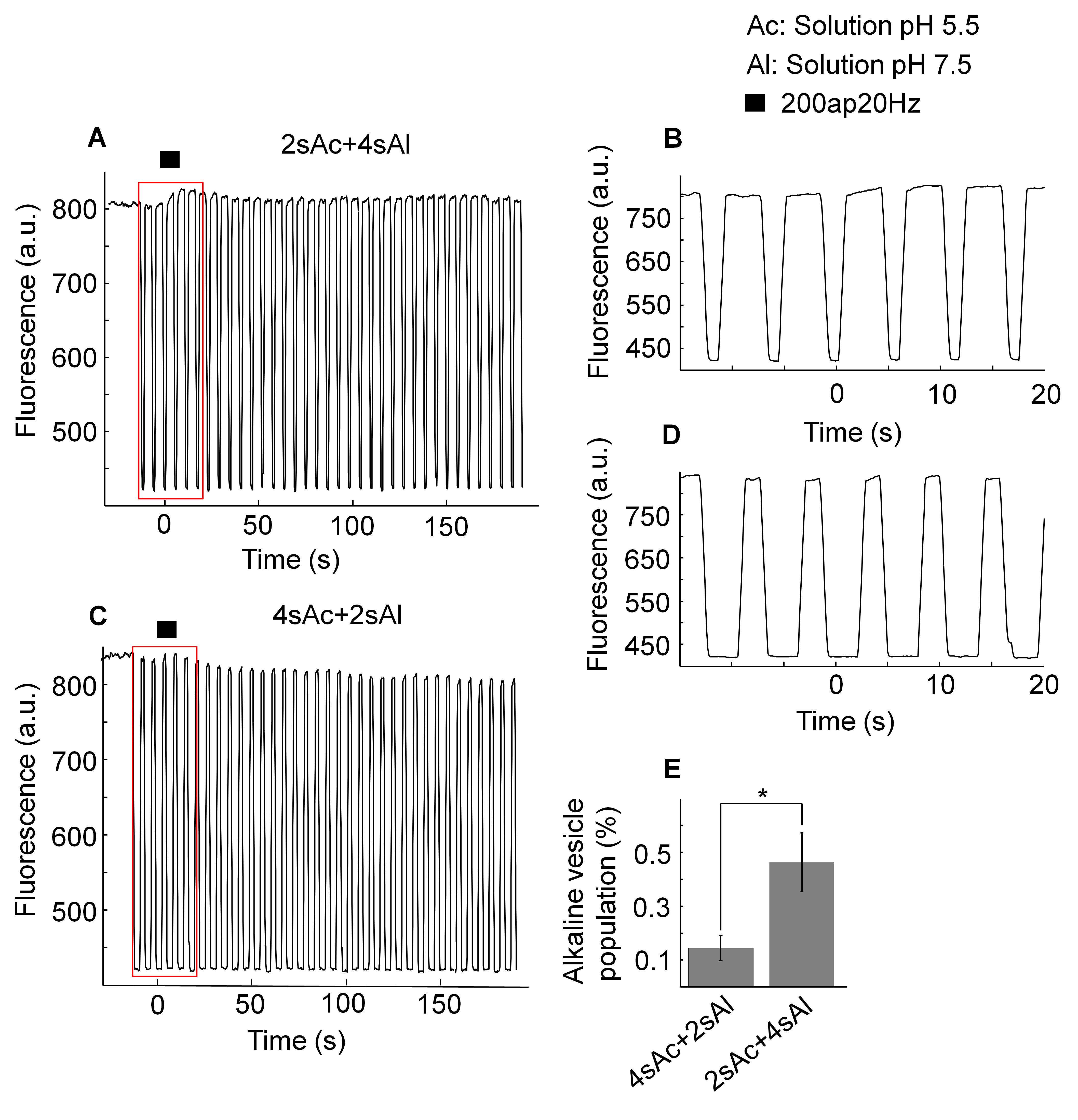

Supplement: Figure S4 — The relative size of the alkaline vesicle population depends on the perfusion interval length. (A) Representative mean fluorescence profile upon pH-cycling with doubled perfusion interval length of pH 7.5. (B) Magnification of the red rectangle marked in (A). (C) Representative mean fluorescence profile upon pH-cycling with doubled perfusion interval length of pH 5.5. (D) Magnification of the red rectangle marked in (C). (E) The relative size of the alkaline vesicle population significantly depends on the perfusion interval length (2 s acid solution +4 s alkaline solution (N = 6, n = 274): 14.55%±0.05 SEM; 2 s alkaline solution +4 s acid solution (N = 6, n = 274): 46.26%±0.11 SEM, p = 0.025). (TIF) [file pone.0102723.s004.tif]

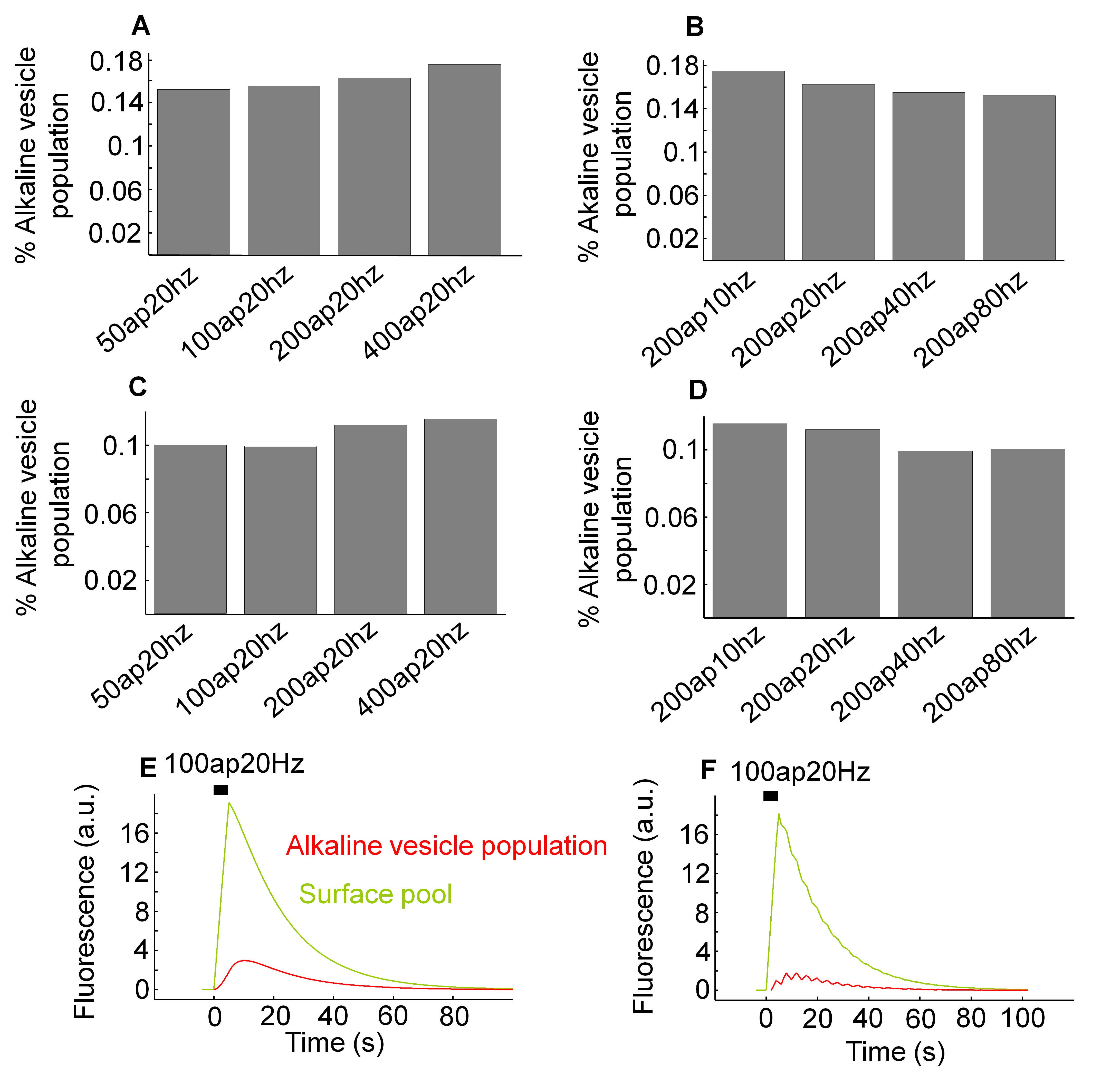

Supplement: Figure S5 — Variation of the relative size of the alkaline vesicle population can be predicted by modeling. For simulation, the time constants for endocytosis and for reacidification were modeled to be invariable to the stimulus condition. (A) Relative size of the alkaline vesicle population depending on the action potential number according to the “ideal” model. The relative alkaline vesicle population size increased with rising number of action potentials. (B) Relative size of the alkaline vesicle population depending on the action potential frequency according to the “ideal” model. The relative alkaline vesicle population size decreased with rising number of action potential frequency. (C) Relative size of the alkaline vesicle population depending on the action potential number according to the “realistic” model. The relative alkaline vesicle population size increased with rising number of action potentials, but was generally smaller compared to the data obtained by the “ideal” model due to underestimation. (D) Relative size of the alkaline vesicle population depending on the action potential frequency according to the “realistic” model. The relative alkaline vesicle population size decreased with rising action potential frequency, but was generally smaller compared to the data obtained by the “ideal” model due to underestimation. (E) Simulated time courses of the surface pool and the alkaline vesicle population according to the “ideal” model. As stimulation paradigms 100 pulses at 20 Hz were assumed. (F) Simulated time courses of the surface pool and the alkaline vesicle population according to the “realistic” model. As stimulation paradigms 100 pulses at 20 Hz were assumed. Vesicles endocytosed upon external pH 5.5 are already acidified and do not contribute to the alkaline pool. (TIF) [file pone.0102723.s005.tif]

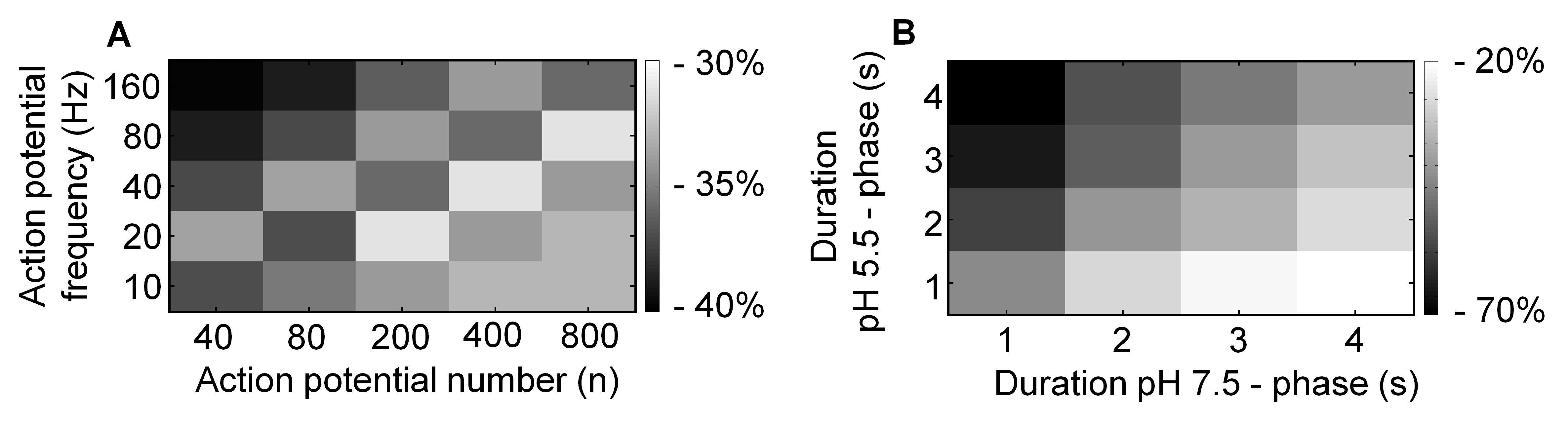

Supplement: Figure S6 — Underestimation effects upon pH-cycling. (A) Underestimation effect upon pH-cycling with balanced perfusion intervals (2 s pH 5.5, 2 s pH 7.5) depending on action potential number and action potential frequency according to the “realistic” model. (B) Underestimation effect upon pH-cycling depending on the acid and alkaline phase length according to the “realistic” model. (TIF) [file pone.0102723.s006.tif]
