# Supplementary material for: Dynamic Properties of the Alkaline Vesicle Population at Hippocampal Synapses
Source: PLoS One. 2014 Jul 31;9(7):e102723. doi: 10.1371/journal.pone.0102723 (PMC4117485; doi:10.1371/journal.pone.0102723)
Supplement: Methods S1 — Simulation of the alkaline vesicle population. (DOCX) [file pone.0102723.s007.docx]

**Methods S1**

**Simulation**

The models we used for data simulation are based on established models [[6](#_ENREF_6),[7](#_ENREF_7)]. They contain the two relevant vesicle pools, the surface pool (S) and alkaline vesicle population (A) (pool of newly endocytosed vesicles) and the processes that contribute to these two pools. Exocytosis (rate constant k_ex_) was modeled as constant during stimulus and proportional to stimulus frequency. Endocytosis (ken) and reacidification (kre) were assumed to be reactions of first order.

**Ideal model:**

During stimulus, the differential equations are:

$$\frac{d}{dt}{S\left( t \right)=-k}_{en}\times S\left( t \right)+k_{ex}$$

$$\frac{d}{dt}{A\left( t \right)=k}_{en}\times S\left( t \right)-k_{re}\times A(t)$$

After stimulus, the differential equations are:

$$\frac{d}{dt}{S\left( t \right)=-k}_{en}\times S\left( t \right)$$

$$\frac{d}{dt}{A\left( t \right)=k}_{en}\times S\left( t \right)-k_{re}\times A(t)$$

The equations were solved symbolically. At the beginning of stimulus, all pool sizes are zero. At the end of stimulus, the respective pool sizes are used as the initial parameters for the post-stimulus phase.

**Realistic model:**

The realistic model uses different equations for the phases with pH 7.5 / pH 5.5 perfusion. Upon pH 7.5 perfusion, the differential equations are equal to the ideal model. Upon pH 5.5 perfusion, the equations reflect the fact that newly endocytosed vesicles are already acidic:

Differential equations during stimulus (pH5.5):

$$\frac{d}{dt}{S\left( t \right)=-k}_{en}\times S\left( t \right)+k_{ex}$$

$$\frac{d}{dt}A\left( t \right)=-k_{re}\times A(t)$$

After stimulus (pH5.5):

$$\frac{d}{dt}{S\left( t \right)=-k}_{en}\times S\left( t \right)$$

$$\frac{d}{dt}A\left( t \right)=-k_{re}\times A(t)$$

The equations were solved symbolically. At the beginning of stimulus, all pool sizes are zero. At the end of each perfusion phase and at the end of stimulus, the respective pool sizes are used as the initial parameters for the next phase. In all simulations (as well as in the experiments) the stimulation began at the beginning of an acid pulse.
