# Supplementary material for: Dynamic Properties of the Alkaline Vesicle Population at Hippocampal Synapses
Source: PLoS One. 2014 Jul 31;9(7):e102723. doi: 10.1371/journal.pone.0102723 (PMC4117485; doi:10.1371/journal.pone.0102723)
Supplement: Text S1 — Performing pH-cycling with VGLUT1-pHluorin. (DOCX) [file pone.0102723.s008.docx]

**Text S1: Performing pH-cycling with VGLUT1-pHluorin.**

To be able to appraise the usability of VGLUT1-pHluorin for pH-cycling, we additionally tried to perform pH-cycling at neurons transfected with VGLUT1-pHluorin. While performing pH-cycling, neurons were electrically stimulated with 200 (N = 7, n = 115) and 100 action potentials (N = 7, n = 96) at 20 Hz. As shown in Figure S2 (A), the mean timecourse of VGLUT1-pHluorin upon pH-cycling exhibited only small fluorescence amplitudes due to the small surface fraction of 2 - 3 % [[11](#_ENREF_11)] but the fluorescence increase upon stimulation was better visible compared to pH-cycling with synapto-pHluorin. Notably, it was possible to reach a stable plateau of fluorescence upon the phases (Figure S2 (B)). However, due to the small fluorescence amplitudes the obtained timecourses were unsuitable for automatic separation of the images obtained at the different pH-values. Nevertheless, we performed further analysis manually, leading to difference images of moderate quality (Figure S2 (C)) and separated mean fluorescence traces of the images obtained upon pH 7.5 (Figure S2 (D)) and 5.5 (Figure S2 (E)) that derived only from single and not averaged images. Afterwards, the relative size of the alkaline vesicle population with respect to the recycling pool was calculated (Figure S2 (F)) and turned out to be not significantly different for the two stimulation paradigms that were tested (p = 0.374). Notably, the relative alkaline vesicle population sizes measured with VGLUT1-pHluorin were lower than when measured with synapto-pHluorin (Figure S2 (G)), but normalized ratios revealed no significant difference in the relative alkaline vesicle population size upon stimulation with 100ap20Hz when measured with synapto-pHluorin or VGLUT1-pHluorin (p = 0.780).
